# Development of a Core Set of Nursing-Sensitive Patient Outcomes in Intensive Care Units: A Delphi Consensus Study

**DOI:** 10.3390/clinpract16050089

**Published:** 2026-04-30

**Authors:** Luciano Cellura, Anna Maria Grugnetti, Stefano Gabriele Scaglia, Attilio Quaini, Silvia Natoli, Giuseppina Grugnetti

**Affiliations:** 1 Emergency and Urgency Department, Foundation Istituto di Ricovero e Cura a Carattere Scientifico (IRCCS), Policlinico San Matteo, 27100 Pavia, Italy; 2 Healthcare Professions Directorate, Foundation Istituto di Ricovero e Cura a Carattere Scientifico (IRCCS), Policlinico San Matteo, 27100 Pavia, Italy; a.grugnetti@smatteo.pv.it (A.M.G.); a.quaini@smatteo.pv.it (A.Q.); g.grugnetti@smatteo.pv.it (G.G.); 3 Women and Maternal–Child Health Department, Foundation Istituto di Ricovero e Cura a Carattere Scientifico (IRCCS), Policlinico San Matteo, 27100 Pavia, Italy; s.scaglia@smatteo.pv.it; 4 Department of Clinical-Surgical, Diagnostic and Pediatric Sciences, University of Pavia, 27100 Pavia, Italy; silvia.natoli@unipv.it; 5 Unit of Pain Therapy Service, Foundation Istituto di Ricovero e Cura a Carattere Scientifico (IRCCS), Policlinico San Matteo, 27100 Pavia, Italy

**Keywords:** nursing-sensitive outcome, nursing quality indicators, intensive care units, consensus study, Delphi method

## Abstract

**Introduction/Aim**: Nursing care constitutes a fundamental determinant of patient outcomes in intensive care units (ICUs); however, the absence of standardised nursing-sensitive indicators constrains the objective evaluation of care quality within this setting. The present study aimed to develop an evidence-informed core set of nursing-sensitive patient outcomes (NSPOs) specific to intensive care nursing. **Methods**: A Delphi consensus study was conducted between September 2023 and February 2025 at the IRCCS Policlinico San Matteo Foundation, Pavia, Italy. The process comprised a preliminary scoping review, followed by two iterative Delphi rounds and a final consensus meeting aimed at refining conceptual domains without modifying item ratings. Thirty-eight ICU nurses evaluated 35 evidence-based NSPOs using a five-point Likert scale. Consensus was defined a priori as ≥75% agreement (scores 4–5), with a minimum response rate of ≥80%. Reliability was assessed using a two-way random-effects Intraclass Correlation Coefficient (ICC). **Results**: Fifteen NSPOs achieved the predefined consensus threshold and demonstrated moderate-to-excellent reliability (ICC = 0.65–0.85). The validated core set was organised into four domains—safety, clinical, functional, and perceptual—reflecting both preventive–technical and holistic dimensions of ICU nursing care. **Conclusions**: This study produced the first ICU-specific evidence-based NSPO core set in Italy, providing a measurable and reproducible framework to support systematic outcome monitoring, and quality improvement in critical care practice.

## 1. Introduction

Nursing care is a fundamental component of critical care, where patients present high clinical complexity and require coordinated multidisciplinary management [1,2]. Despite its central role, the specific contribution of nursing to patient outcomes remains difficult to quantify due to the limited availability of standardised and reliable indicators [2,3,4].

Outcome assessment remains predominantly medically oriented, reflecting a predominantly physician-oriented framework that may insufficiently capture the multidimensional nature of care [5]. As a result, nursing care quality, despite being a central dimension of healthcare quality and Clinical Governance [6,7,8], is still inadequately represented in routine evaluation systems [9].

Nursing-sensitive patient outcomes (NSPOs) were introduced to identify patient and family outcomes directly attributable to nursing care. These measures support accountability and evidence-informed decision-making [10,11,12]. These outcomes are conceptually rooted in models linking nursing structures, processes, and outcomes [7,13]; however, their application in ICU settings remains limited. The literature on nursing-related outcomes shows substantial heterogeneity in measurement instruments, particularly for symptom burden and severity, highlighting persistent problems in standardisation [14]. Although nursing-related variables such as educational preparation, skill mix, and organisational context have been associated with patient outcomes [15,16,17], the operationalisation of NSPOs remains inconsistent [11,16,17,18], and consensus on which outcomes are specifically attributable to nursing care is still lacking [19,20].

To date, research has focused mainly on broad multifactorial outcomes, such as mortality, length of stay, infections, falls, and medication errors [20,21], which are strongly influenced by factors beyond nursing care. Conversely, outcomes more closely related to nursing practice, including hygiene, symptom management, comfort, and psychological well-being, remain underrepresented despite their clinical relevance [22]. In ICUs, this limitation is further intensified by the interaction of clinical, organisational, and technological determinants, which makes outcome attribution particularly challenging [23,24]. The absence of validated frameworks [3], variability in ICU nursing competencies [25,26], and the lack of systematic measurement tools further hinder the integration of NSPOs into quality monitoring and Clinical Governance [9].

Therefore, two major gaps persist—the absence of a shared, context-specific NSPO framework for ICU settings and the lack of consensus on clinically meaningful outcomes sensitive to nursing care. In this context, consensus-based methodologies are essential for translating heterogeneous evidence into structured and clinically applicable frameworks [14]. This study aims to develop a consensus-based core set of NSPOs for an Italian ICU, integrating evidence synthesis and structured expert consensus to define a concise and operational set of clinically relevant outcomes for critical care nursing evaluation.

## 2. Materials and Methods

### 2.1. Design

A multi-round Delphi technique was employed to develop a consensus-based set of NSPOs, in accordance with CREDES guidelines [27], ACCORD standards [28], and the COMET Handbook (version 1.0) [29]. This approach was selected to achieve structured consensus among expert nurses in intensive and critical care.

The Delphi method assumes that collective expert judgement provides more robust estimates than individual opinions [30]. Through iterative rounds with controlled feedback, it enables progressive convergence of expert views while allowing formal assessment of consensus and response stability.

A formal protocol was developed a priori and applied to both the scoping review and Delphi phases, ensuring methodological transparency and reproducibility [31,32,33,34,35,36]. The protocol predefined all key components, including eligibility criteria, search strategy, study selection, data extraction, panel recruitment, consensus thresholds, stability criteria, and the number of rounds. Although not preregistered, methodological bias was mitigated through strict a priori specification and adherence to recognised reporting standards [27,28,29].

Consensus was operationalised using predefined quantitative thresholds, enabling objective identification of agreement. In parallel, response stability across rounds was assessed to evaluate the consistency of expert judgements over time. These criteria were established to prevent post hoc decision-making and ensure methodological consistency.

The Delphi process was specifically tailored to the ICU context to evaluate NSPOs in terms of nursing attribution, defined as the extent to which an outcome is directly influenced by nursing care processes. Anonymity and controlled feedback minimised social desirability bias and dominance effects, supporting independent judgement and balanced participation.

**Figure 1:**
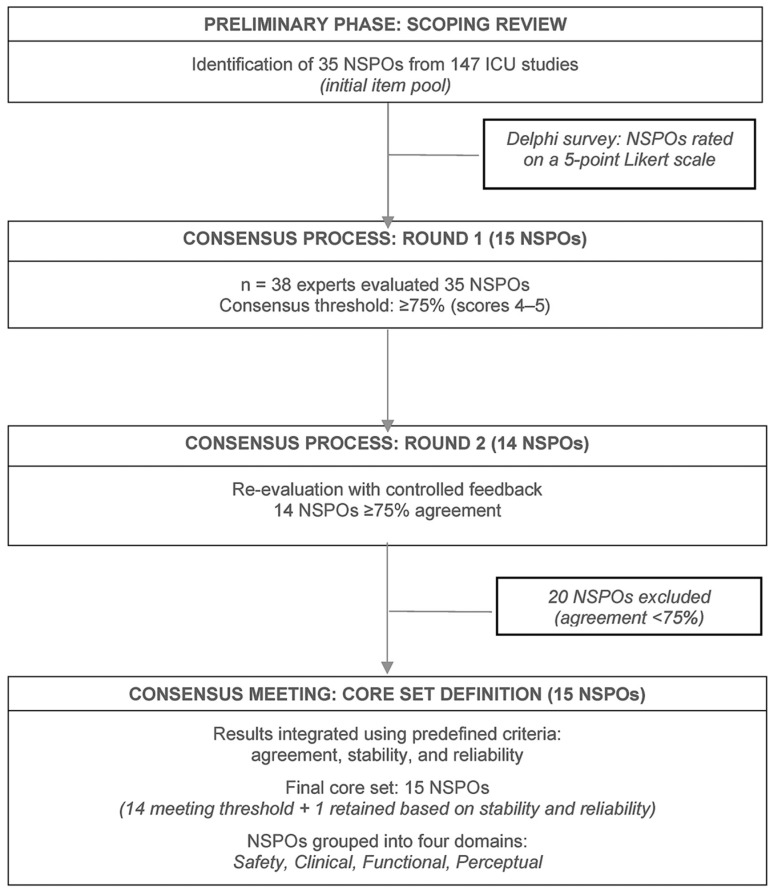
Flow chart of the overall study design. The figure outlines the sequential refinement of NSPOs from evidence synthesis to final consensus. A two-round Delphi process was conducted using predefined criteria of agreement, stability, and reliability, leading to a final core set of 15 NSPOs.

This design ensured methodological rigour, reproducibility, and systematic integration of expert knowledge throughout the consensus process (Figure 1).

### 2.2. Consensus Process

The consensus process followed a structured two-round Delphi design with controlled feedback, enabling progressive refinement of expert judgement and convergence on core NSPOs.

A preliminary scoping review (September 2023–September 2024) systematically mapped the literature from 1996—the year of the first formal definition of NSPOs [10,37]—to the present, ensuring conceptual saturation and informing item generation. Based on this phase, 35 candidate NSPOs were included as uncategorised items in the Delphi survey.

Two rounds were conducted between November 2024 and February 2025. Participants rated each item on a five-point Likert scale, assessing relevance within the ICU nursing context. After each round, aggregated results and anonymised feedback were provided, allowing participants to reconsider responses in light of group trends while minimising dominance and conformity bias.

Consensus was defined as ≥75% agreement (scores 4–5) [29], consistent with methodological recommendations (70–80%) balancing rigour and inclusiveness [38,39,40,41]. Stability was assessed in Round 2 using predefined criteria—unchanged median (ΔMedian = 0) and a stable or reduced interquartile range (IQR)—reflecting convergence of expert judgement.

All methodological parameters—including number of rounds, consensus thresholds, and feedback procedures—were established a priori to ensure transparency and prevent post hoc bias. As stability criteria were met after Round 2, no additional rounds were conducted, indicating that further iterations were unlikely to yield meaningful changes in consensus.

### 2.3. Preliminary Phase: Literature Search Strategy

A scoping review was conducted in accordance with the Arksey and O’Malley framework [42] to systematically map the literature on NSPOs in ICU settings and support the generation of candidate NSPOs for the subsequent Delphi process.

The search strategy combined MeSH terms and free-text keywords related to NSPOs, intensive care, and quality indicators using Boolean operators (Table S1). Searches were performed in MEDLINE (PubMed), CINAHL, Embase, and Scopus.

Eligibility criteria were defined a priori and included primary and secondary studies investigating NSPOs, as defined by Maas et al. [10], in adult ICU populations (≥18 years). Only studies explicitly linking nursing interventions or metrics to patient outcomes were included. Articles were limited to full-text publications in English.

Study selection followed a two-stage process (title/abstract screening and full-text review), conducted independently by two reviewers (LC, SGS) using Rayyan software (v.1.4.3; Rayyan Systems Inc., Cambridge, MA, USA) [43]. Discrepancies were resolved through discussion (agreement > 80%). Additional studies were identified through citation tracking and Google Scholar searches (Figure S1).

All procedures—including eligibility criteria, screening, and data extraction—were predefined and systematically applied [44]. Data were extracted into a structured database (Microsoft Corporation, Redmond, WA, USA; Mac version 15.26), including study characteristics, populations, nursing variables, and outcomes.

Given the purpose of this preliminary phase—to support the generation of candidate NSPOs for the Delphi process—no formal assessment of methodological quality was undertaken.

Extracted data were synthesised through a structured multi-stage process involving verbatim coding, semantic clustering, and conceptual mapping. This approach enabled the generation of a coherent set of candidate NSPOs and ensured conceptual saturation.

### 2.4. Survey Characteristics

A digital survey was developed using Google Forms^®^ (Google LLC, Mountain View, CA, USA) [45] and underwent expert review to ensure content validity, clarity, and alignment with study objectives. The instrument was independently evaluated by an academic expert for precision and coherence.

The final survey comprised three sections. The first provided study background, objectives, procedural details of the Delphi rounds, and information on confidentiality, data protection, and informed consent, ensuring compliance with ethical standards. Participants were informed that item wording would remain unchanged across rounds to preserve methodological consistency.

The second section collected demographic data, including gender, professional education, and years of ICU experience. These variables were predefined to support subgroup analyses and transparent reporting of panel characteristics, in line with CREDES and ACCORD recommendations [27,28].

The third section included 35 NSPO items derived from the preliminary phase, each presented as a statement and rated on a five-point Likert scale (1 = strongly disagree to 5 = strongly agree) [46]. A neutral midpoint was retained to reduce forced-choice bias. Item order and scale structure were kept constant across rounds to ensure response stability.

The Likert format enabled quantification of agreement and facilitated the evaluation of consensus in subsequent Delphi rounds. All survey features, including rating strategy and administration procedures, were predefined to ensure methodological transparency and reproducibility.

### 2.5. Participants: Composition of the Expert Panel

Participants were recruited from the ICU nursing staff of the Fondazione IRCCS Policlinico S. Matteo (Pavia, Italy) using an a priori sampling strategy designed to ensure a 1:1 ratio between two stakeholder groups, thereby balancing clinical and specialist expertise. The final panel comprised 38 experts selected from a workforce of 80 eligible nurses. All eligible participants were invited via institutional email, with voluntary participation and no incentives, ensuring independence from managerial or clinical hierarchies.

Although conducted within a single institution, the sampling strategy aimed to maximise internal heterogeneity by including nurses with diverse clinical experience, educational backgrounds, and professional roles, thereby strengthening the internal validity of the consensus process.

Group 1 (Intensive Care Nursing Experts) included nurses with ≥5 years of ICU experience, ensuring advanced clinical competence in managing critically ill patients [47]. Group 2 (Specialist Nurses in Critical Care) comprised nurses holding a postgraduate (Level I) qualification in critical care with ≥1 year of ICU experience, reflecting advanced theoretical and clinical training [47].

The inclusion of participants with varying experience levels, including early-career nurses, was intentional to reflect the real-world ICU workforce. While this may have favoured more observable or protocol-driven outcomes, it enhanced the practical relevance of the consensus. Overall, the panel composition integrated complementary clinical and specialist perspectives, strengthening the contextual validity of NSPO evaluation [48].

In Delphi methodology, panel size is determined pragmatically based on expertise rather than statistical power [27,28]. Previous studies report panels ranging from 10 to 40 participants—thus, the inclusion of 38 experts was considered methodologically appropriate to ensure stable and credible consensus outcomes [49].

### 2.6. Number of Rounds

The number of Delphi rounds was predefined at two, with provision for a third if further refinement was required. This decision reflects methodological evidence indicating that additional iterations beyond two to three rounds yield limited gains in consensus while increasing response burden [29,49,50].

Round 1 captured initial expert judgements on the relevance of candidate NSPOs, whereas Round 2 incorporated controlled statistical feedback, enabling participants to reassess responses based on aggregated group results and promoting convergence of opinion.

The decision to limit the process to two rounds was guided by stability criteria rather than procedural convenience. Stability was defined as unchanged median values (ΔMedian = 0) and stable or reduced interquartile ranges (IQRs), consistent with established Delphi convergence standards.

These criteria were fully achieved after Round 2, with stable medians and reduced dispersion across items, indicating convergence of expert judgement. Consequently, a third round was not conducted, as further iterations were unlikely to produce meaningful changes in consensus.

### 2.7. Data Analysis

In accordance with COMET standards, a minimum response rate of ≥80% per stakeholder group was considered acceptable for each Delphi round [29]. Overall and group-specific response rates were calculated to assess participation consistency. All analytical procedures—including consensus thresholds, stability criteria, and reliability metrics—were predefined a priori to ensure transparency and reproducibility.

Descriptive statistics (absolute frequencies and percentages) were used to summarise Likert-scale responses and quantify agreement for each NSPO, defined as the proportion of ratings ≥ 4. Temporal stability between rounds was assessed by reconstructing item-level distributions to calculate medians and interquartile ranges (IQRs). Stability was defined as unchanged medians (ΔMedian = 0) with stable or reduced IQRs, in line with established Delphi convergence criteria.

Inferential analyses explored differences across stakeholder groups using the Kruskal–Wallis nonparametric test, with significance set at *p* < 0.05. Reliability of expert ratings was assessed using the Intraclass Correlation Coefficient two-way random-effects (ICC 2.1) absolute agreement. Although Likert data are ordinal, their treatment as quasi-interval measures is supported in agreement studies, and ICC is considered appropriate for Delphi designs [50,51,52]. Reliability was interpreted as poor (<0.50), moderate (0.50–0.74), good (0.75–0.89), or excellent (≥0.90) [52].

Survey data were exported from Google Forms^®^ and organised in Microsoft Excel (XLS), with rows representing participants and columns representing NSPO items. Analyses were conducted using Microsoft Excel 2016 (Mac version 15.26) and JASP (version 0.19.3; University of Amsterdam, Amsterdam, The Netherlands), ensuring reproducibility and traceability of statistical procedures.

### 2.8. Ethical Considerations

This study was approved by the IRCCS Policlinico San Matteo Foundation (Protocol No. 0026012/24) and conducted in accordance with institutional and national regulations.

Participants received detailed information on study objectives, procedures, and data management, and provided written informed consent. Participation was voluntary, with the right to withdraw at any time without consequences.

Data were collected anonymously and analysed in aggregated form to prevent individual identification. All records were securely stored in password-protected systems accessible only to authorised researchers.

Data management complied with the General Data Protection Regulation (EU GDPR No. 679/2016) and relevant Italian legislation (Legislative Decrees No. 196/2003 and No. 101/2018) [53].

The study was conducted in full respect of professional autonomy, confidentiality, and ethical integrity, without interfering with routine clinical practice.

## 3. Results

### 3.1. Preliminary Phase Results

A total of 10,738 records were identified; 6642 records were removed following duplicate removal and the application of predefined filters, and 3923 were excluded during title/abstract screening. Of 187 full-text articles assessed, 144 met the inclusion criteria. Three additional studies were identified through supplementary searches, yielding a final sample of 147 studies (Figure S1; Table S2).

### 3.2. Outcome Mapping and Synthesis Process

From 147 studies, 420 raw outcome expressions were extracted and reduced through a structured multi-stage process involving semantic clustering (*n* = 110) and deductive mapping to Doran’s domains [16] (Figure S2), followed by conceptual consolidation. This process generated 35 distinct NSPOs, ensuring conceptual coherence and saturation (Table S3).

### 3.3. Delphi Survey Results

Of 38 enrolled participants, 37 completed Round 1, and 31 completed both rounds (overall response rate: 82%), meeting predefined thresholds. Panel characteristics (Table 1) indicate a predominance of female nurses (61%) with postgraduate education (58%) and ≤5 years ICU experience, consistent with workforce composition. The predefined balance between clinical and specialist profiles was maintained.

No withdrawals occurred, and incomplete responses were managed according to predefined procedures, ensuring consistency across rounds.

### 3.4. Round 1

**Figure 2:**
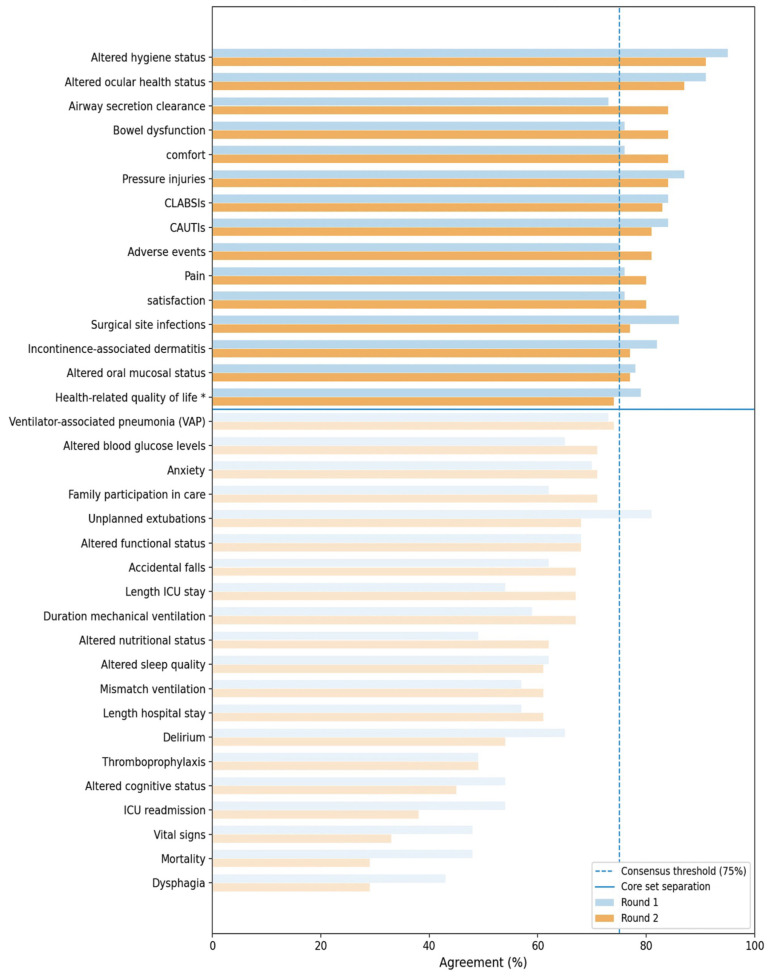
Agreement distribution across Delphi rounds. Bars represent agreement percentages in Round 1 and Round 2. The dashed vertical line indicates the predefined consensus threshold (≥75%). NSPOs are ordered by Round 2 agreement and grouped according to inclusion in the final core set (*n* = 15), highlighting progressive convergence and differentiation between included and excluded outcomes. * Retained based on stability (ΔMedian = 0; IQR = 0) and reliability criteria, despite not reaching the Round 2 consensus threshold.

Figure 2 presents the distribution of agreement across Delphi rounds, illustrating progressive convergence of expert judgement and the distinction between outcomes included in the final core set and those excluded.

In Round 1 (response rate: 97%), 15 of 35 NSPOs reached consensus (≥75% agreement). The highest agreement was observed for hygiene (95%), ocular health (91%), pressure injuries (87%), and surgical site infections (86%), all characterised by low dispersion (IQR = 0), indicating early convergence.

In contrast, system-level outcomes—mortality (48%), length ICU stay (54%), and readmission (54%)—showed lower agreement and greater variability, reflecting their multifactorial nature and limited nursing attribution.

These findings demonstrate a clear distinction between high attribution (care-sensitive) and low attribution (system-level) outcomes, supporting the specificity of NSPOs as indicators of nursing performance.

### 3.5. Round 2

Round 2 (response rate: 84%) confirmed convergence of expert judgement. Fourteen indicators met the predefined ≥75% agreement threshold. One indicator—health-related quality of life—did not reach the agreement threshold in Round 2 (74%), but exhibited a stable distributional pattern across rounds. Median values remained unchanged (Median = 4; ΔMedian = 0), with no dispersion (IQR = 0), and reliability fell within the good range (ICC = 0.81). This indicator had previously achieved consensus in Round 1 (79%).

In parallel, several indicators demonstrated increased agreement and improved reliability, including airway secretion clearance (ICC = 0.79) and nutritional status (ICC = 0.78), whereas others showed reduced agreement (e.g., ICU readmission, ICC = 0.74). Full dataset verification confirmed consistency between agreement levels, stability metrics, and reliability coefficients (Table S4).

These findings indicate progressive convergence of expert judgement and refinement of NSPO classification boundaries.

### 3.6. Consensus Meeting and Core Set Definition

The integration of agreement (≥75%), stability (ΔMedian = 0; IQR convergence), and reliability (ICC ≥ 0.65) resulted in a final core set of 15 NSPOs (Table 2). While 14 indicators met the agreement threshold in Round 2, health-related quality of life was retained on the basis of its longitudinal consistency across rounds.

Specifically, the indicator maintained a stable central tendency (Median = 4; ΔMedian = 0) with no dispersion (IQR = 0) and demonstrated good reliability (ICC = 0.81). The slight reduction in agreement between Round 1 (79%) and Round 2 (74%) was not associated with changes in response distribution and is therefore interpretable as a marginal redistribution rather than a substantive shift in expert judgement. Within this context, its inclusion reflects the consistent application of the predefined analytical framework, based on the integration of multiple parameters rather than strict adherence to a single threshold criterion.

Health-related quality of life represents a central perceptual outcome within the NSPO construct, capturing patient-centred dimensions intrinsically linked to nursing care. Its inclusion ensures structural completeness of the perceptual domain within the final core set.

The final set spans four domains (Table 2)—safety, clinical, functional, and perceptual—and is consistent with the established NSPO taxonomy. Safety indicators showed the highest levels of agreement (81–93%), particularly for preventable complications (e.g., pressure injuries, CAUTIs, CLABSIs), reflecting strong nursing attribution. Clinical and functional domains describe physiological management and fundamental care processes, while perceptual outcomes (e.g., satisfaction, health-related quality of life, comfort) reflect core dimensions of patient experience.

Overall, the resulting core set constitutes a preliminary, standardised, and clinically relevant framework for ICU nursing outcome evaluation, supported by high levels of agreement, response stability, and acceptable reliability [54,55,56].

## 4. Discussion

This study developed a consensus-based core set of 15 NSPOs for ICU settings through a structured two-round Delphi process. The resulting framework, supported by predefined criteria of agreement, stability (ΔMedian = 0; IQR convergence), and reliability (ICC ≥ 0.65), may be interpreted as a preliminary yet conceptually grounded and operationally oriented tool for evaluating nursing care quality in critical care environments. Given the single-centre design and the exploratory nature of the consensus process, further validation in multicentre settings is warranted to assess its transferability and applicability across diverse clinical and organisational contexts [57].

The findings highlight a clear distinction between outcomes characterised by high versus low nursing attribution. Indicators achieving early and stable consensus—such as hygiene status, pressure injuries, CAUTIs, CLABSIs, and incontinence-associated dermatitis—are closely linked to fundamental care processes and the prevention of care-related complications, widely recognised as central to ICU nursing practice [55]. These outcomes consistently demonstrated high agreement and low variability, supporting convergence of expert judgement and their sensitivity to nursing care processes.

Within this context, the variation in the number of indicators across rounds (15 in Round 1, 14 in Round 2, and 15 in the final set) reflects sensitivity to predefined consensus thresholds rather than inconsistency in the decision-making process. This aspect was addressed through an integrated evaluation of agreement, stability (ΔMedian = 0; IQR), and reliability (ICC), enabling the retention of outcomes characterised by stable central tendency and acceptable reliability [54]. The observed stability of responses across rounds—reflected by unchanged medians and reduced or stable interquartile ranges—together with moderate-to-good ICC values, supports the internal consistency of expert judgements and alignment with the predefined analytical strategy. The convergence of agreement, dispersion, and reliability measures reinforces the internal coherence of the dataset and the transparency of the consensus process [27,56,58].

Conversely, outcomes such as mortality, ICU length of stay, and readmission did not reach consensus and were associated with greater variability in expert ratings. This pattern likely reflects their intrinsically multifactorial nature, shaped by the interaction of clinical decision-making, organisational factors, and patient-level determinants. Their exclusion should therefore be interpreted as a clarification of attribution boundaries rather than a limitation of the study, in line with previous Delphi research [54,55,56]. This distinction contributes to a more precise delineation of nursing-sensitive outcomes within complex multidisciplinary environments.

When considered in relation to the international literature, these findings show substantial convergence with existing conceptualisations of nurse-sensitive outcomes, while offering a more explicitly operational perspective. Yang et al. identified 20 indicators structured across structure, process, and outcome domains [59], whereas Chrusch and Martin proposed 22 indicators across six dimensions, with particular emphasis on safety and patient-centred outcomes [60]. Similarly, the present study confirms the centrality of safety-related and perceptual domains, particularly in relation to preventable complications and patient experience [61]. This alignment situates the findings within a broader evidence base and supports their conceptual relevance.

This interpretation is consistent with evidence demonstrating that nursing care exerts a measurable impact on clinical outcomes [62,63], particularly those related to care processes and clinical surveillance. Higher levels of nurse staffing have been associated with reductions in adverse events such as pressure injuries, infections, and medication errors, as well as improvements in mortality outcomes, underscoring the sensitivity of these indicators to variations in nursing care quality [62,64,65,66].

From a conceptual perspective, Delphi-derived frameworks should be interpreted as structured expert-based guidance rather than formal clinical practice guidelines, particularly in contexts characterised by heterogeneous or limited empirical evidence [58]. This distinction is essential to appropriately position NSPO frameworks within the hierarchy of evidence and to avoid overinterpretation of consensus-based findings. The primary contribution of this study lies in the operationalisation of NSPOs through explicit attribution criteria, advancing beyond descriptive classifications toward measurable and clinically meaningful constructs.

From a clinical and organisational perspective, the proposed core set offers a structured and potentially applicable framework for integrating NSPOs into ICU quality monitoring systems, including clinical audits, benchmarking activities, and performance dashboards. By prioritising outcomes with high nursing attribution, it may contribute to improving both sensitivity and specificity in the evaluation of nursing care quality. Potential applications include integration into Nursing Minimum Data Sets (NMDSs) [67] and electronic health records, supporting systematic data collection and enhancing the visibility of nursing contributions. However, implementation requires standardised definitions, interoperable information systems, and targeted staff training, which remain areas for further methodological and organisational development.

The exclusion of traditionally used outcomes, such as mortality and length of stay, highlights the need to move beyond generic performance metrics toward more specific and nursing-sensitive measures. If confirmed in broader contexts, this perspective may contribute to refining conventional approaches to outcome evaluation and support a more nuanced understanding of professional accountability within complex care environments.

### Strengths and Limitations

This study presents several limitations. The single-centre design may have introduced institutional and cultural influences that could have shaped consensus patterns, thereby limiting external validity and transferability despite panel heterogeneity [68]. The inclusion of a substantial proportion of nurses with less than five years of ICU experience may have oriented consensus toward more observable and protocol-driven aspects of care, highlighting the need for further investigation into the role of expertise distribution in Delphi panels. Participant attrition represents an inherent limitation of Delphi studies; however, response rates remained ≥ 80% across rounds [29], with no significant differences observed between completers and non-completers, supporting panel stability.

Although a formal methodological protocol was developed and applied a priori, it was not preregistered. Consequently, transparency and reproducibility are inherently less robust than in formally preregistered designs, and the risk of methodological and reporting bias cannot be fully excluded.

Despite these limitations, the study demonstrates notable strengths. Methodological rigour was ensured through adherence to established frameworks [27,28,29] and the a priori definition of consensus thresholds, stability criteria, and analytical procedures. The combined use of agreement, dispersion, and reliability measures enabled a robust, multidimensional assessment of consensus, strengthening internal validity and overall credibility.

## 5. Conclusions

This study developed a consensus-based core set of 15 ICU-specific NSPOs through a structured two-round Delphi process informed by evidence synthesis. The resulting framework represents a preliminary, conceptually grounded, and operationally oriented approach to capturing outcomes directly attributable to nursing care in critical care settings.

The identified core set spans Doran’s four conceptual domains—safety, clinical, functional, and perceptual [16]—and incorporates both prevention-oriented and patient-centred dimensions, reflecting the complexity of ICU nursing practice, where clinical stability, prevention, and patient experience converge. The findings contribute to clarifying the distinction between nursing-attributable and system-level outcomes, supporting a shift from generic performance metrics toward more specific and nursing-sensitive measures, with potential implications for improving interpretability in outcome evaluation within multidisciplinary environments.

Given the single-centre design and the consensus-based nature of the methodology, these findings should be interpreted with caution. While agreement, stability, and reliability metrics support the internal consistency of the results, further validation in multicentre settings with larger and more heterogeneous samples is required to assess generalisability and applicability across contexts.

From an applied perspective, the proposed NSPO core set may represent a potentially scalable framework with relevance for integration into Nursing Minimum Data Sets (NMDSs) [67], ICU quality monitoring systems, and digital clinical information platforms. However, such applications remain contingent on further validation, standardisation of definitions, and alignment with existing information infrastructures.

Future research should prioritise multicentre validation, feasibility testing in routine clinical settings, and integration into electronic health records to support sustainability and broader applicability across healthcare contexts.

## Figures and Tables

**Table 1:** Panel characteristics.

Category	Round 1 (*n* = 38) November–December 2024 *n* (%)	Round 2 (*n* = 31) January–February 2025 *n* (%)
Participation rate	37 (97)	31 (84)
Gender		
Males	15 (40)	12 (39)
Females	22 (60)	19 (61)
Professional education level		
Bachelor’s degree	19 (51)	13 (42)
Postgraduate/Master degree	18 (49)	18 (58)
Years of ICU experience		
0–5 years	14 (38)	14 (45)
6–10 years	10 (27)	10 (32)
≥11 years	13 (35)	7 (23)

**Table 2:** Final Core Set of NSPOs.

Domain	NSPOs	ΔMedian (R1–R2)	ICC	IQR (R1/R2)	Inclusion Criterion
Safety	Adverse events	0	0.78	0/0	Consensus
	Pressure injuries	0	0.82	0/0	Consensus
	Catheter-associated urinary tract infections (CAUTIs)	0	0.72	0/0	Consensus
	Surgical site infections	0	0.66	0/0	Consensus
	Central line-associated bloodstream infections (CLABSIs)	0	0.68	0/0	Consensus
	Incontinence-associated dermatitis	0	0.69	0/0	Consensus
Clinical	Airway secretion clearance	0	0.79	0/0	Consensus
	Pain	0	0.83	0/0	Consensus
Functional	Bowel dysfunction	0	0.80	0/0	Consensus
	Altered oral mucosal status	0	0.71	0/0	Consensus
	Altered ocular health status	0	0.70	0/0	Consensus
	Altered hygiene status	0	0.72	0/0	Consensus
Perceptual	Comfort	0	0.76	0/0	Consensus
	Satisfaction	0	0.85	0/0	Consensus
	Health-related quality of life *	0	0.81	0/0	Stability and reliability

Notes: Consensus was defined as ≥75% agreement (ratings of 4–5 on a 5-point Likert scale, Round 2). Stability was defined as ΔMedian = 0; IQR convergence and reliability ICC ≥ 0.65. * Retained based on predefined criteria despite not reaching the Round 2 consensus threshold.

## Data Availability

The data supporting this study are available from the corresponding author upon reasonable request.

## Supplementary Materials

The following supporting information can be downloaded at https://www.mdpi.com/article/10.3390/clinpract16050089/s1. Figure S1: Preferred Reporting Items for Systematic Review and Meta-Analyses PRISMA flow diagram. PRISMA flow diagram [69] illustrating the study selection process and inclusion criteria for the scoping review. Figure S2: Mapping and synthesis process of nursing-sensitive patient outcomes (NSPOs) extracted from the scoping review. The figure summarises the multi-step analytical procedure—verbatim extraction, semantic clustering, domain mapping, and conceptual consolidation—used to derive the NSPO set evaluated in the Delphi rounds. Table S1: Search strategy and research strings used for the scoping review (Preliminary Round), detailing databases, time frames, and keywords applied in accordance with PRISMA-ScR guidelines [70]. Table S2: Data summary tables reporting the main characteristics and variables extracted from all studies included in the preliminary round scoping review. Table S3: Extended set of nursing-sensitive patient outcomes (NSPOs) with conceptual origins and typical metrics. The table presents the complete list of NSPOs derived from the included studies and outlines the synthesis approach used to formulate the extended set of 35 NSPOs included in the Delphi process. Table S4: Complete dataset of the Delphi consensus process, including item-level responses and agreement percentages for each NSPOs across both Delphi rounds. The structured dataset provided the basis for mapping, classifying, and synthesising NSPOs.
