# Supplementary material for: Development of a Core Set of Nursing-Sensitive Patient Outcomes in Intensive Care Units: A Delphi Consensus Study
Source: Clin Pract. 2026 Apr 30;16(5):89. doi: 10.3390/clinpract16050089 (PMC13206322; doi:10.3390/clinpract16050089)

Figure S1. Preferred Reporting Items for Systematic Review and Meta-Analyses PRISMA flow diagram [69].

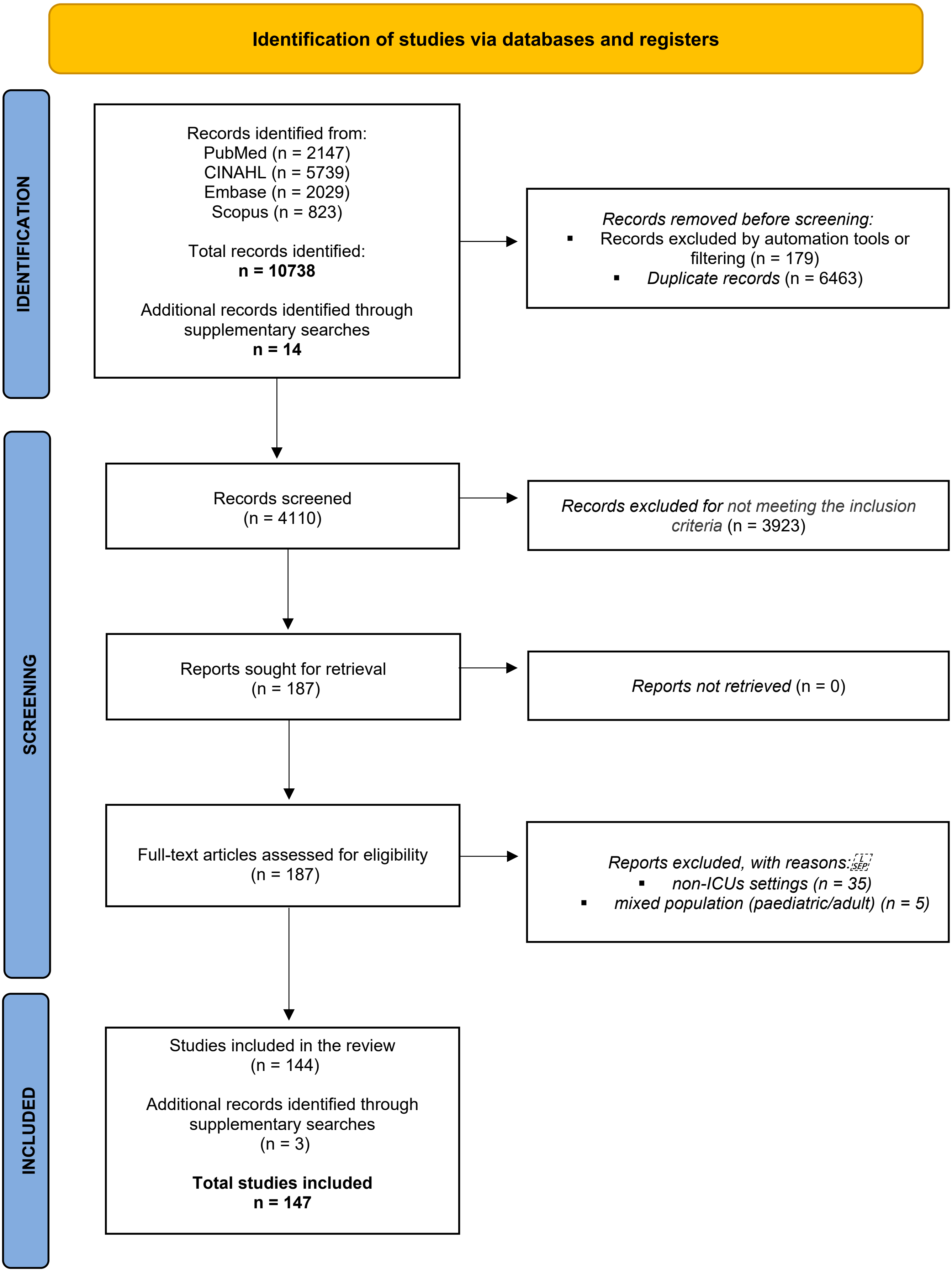

Supplement: Supplementary file 1 [file clinpract-16-00089-s001.zip › Figure S1. Preferred Reporting Items for Systematic Review and Meta-Analyses PRISMA flow diagram..pdf]
