# Supplementary material for: Development of a Core Set of Nursing-Sensitive Patient Outcomes in Intensive Care Units: A Delphi Consensus Study
Source: Clin Pract. 2026 Apr 30;16(5):89. doi: 10.3390/clinpract16050089 (PMC13206322; doi:10.3390/clinpract16050089)
Supplement: Supplementary file 1 [file clinpract-16-00089-s001.zip › Figure S2. Mapping and synthesis process of Nursing Sensitive Patient Outcomes (NSPOs) extracted from the scoping review..pdf]

Figure S2. Mapping and synthesis process of nursing-sensitive patient outcomes (NSPOs) extracted from the scoping review.

Stage1 - Verbatim Extraction

Two reviewers independently transcribed every outcome label, sub-outcome and metric reported in each study, yielding n = 420 raw outcome expressions.

Stage 2 - Semantic Clustering

Similar or synonymous terms were grouped according to lexical overlap and conceptual meaning, forming n = 110 preliminary clusters.

Stage 3 - Deductive Domain Mapping

Each cluster was assigned to one of Doran's four domains (safety, clinical, functional, perceptual) [16], ensuring alignment with a recognised conceptual framework.

Stage 4 - Conceptual Reduction (Axial Synthesis)

Clusters were refined through iterative merging, removal of redundancies, and terminology harmonisation, resulting in 35 distinct nursing-sensitive patient outcomes (NSPOs).

Legend. The figure depicts the process through which all outcome expressions reported in the 147 studies included (**Table S2.** Data Summary) in the scoping review were transformed into a coherent set of 35 nursing-sensitive patient outcomes. Following verbatim extraction by two reviewers (420 expressions), outcomes were grouped into semantic clusters and mapped to Doran's four conceptual domains [16]. A final stage of conceptual consolidation eliminated redundancies and harmonised terminology, resulting in 35 distinct NSPOs.
