# Supplementary material for: Development of a Core Set of Nursing-Sensitive Patient Outcomes in Intensive Care Units: A Delphi Consensus Study
Source: Clin Pract. 2026 Apr 30;16(5):89. doi: 10.3390/clinpract16050089 (PMC13206322; doi:10.3390/clinpract16050089)
Supplement: Supplementary file 1 [file clinpract-16-00089-s001.zip › Table S1. Search Strategy..pdf]

Table S1. Search Strategy.

	Last Updated on 30 May 2024	
MEDLINE (PubMed)	*(((“Critical Illness”[Text Word] OR “Critically ill patients”[Text Word]) AND (“Nurse Sensitive Outcomes”[Text Word] OR “Nursing-sensitive indicators”[Text Word] OR “Critical Care Outcomes”[MeSH Terms] OR “Patient Outcome Assessment”[MeSH Terms] OR “Treatment Outcome”[MeSH Terms]) AND (“nursing”[MeSH Subheading] OR “Critical Care”[Text Word] OR “Critical Care Nursing”[MeSH Terms] OR “Intensive Care Nursing”[All Fields] OR “Intensive Care Units”[MeSH Terms] OR “Emergency Nursing”[MeSH Terms] OR “Advanced Practice Nursing”[MeSH Terms]))*	n = 2147
CINAHL (EBSCO)	*(((“Critical Illness” OR “Critically ill patients” AND “Nurse Sensitive Outcomes”OR “Nursing-sensitive indicators”[Text Word] OR “Critical Care Outcomes”[MeSH Terms] OR “Patient Outcome Assessment”[MeSH Terms] OR “Treatment Outcome”[MeSH Terms]) AND (“nursing”[MeSH Subheading] OR “Critical Care”[Text Word] OR “Critical Care Nursing”[MeSH Terms] OR “Intensive Care Nursing”[All Fields] OR “Intensive Care Units”[MeSH Terms] OR “Emergency Nursing”[MeSH Terms] OR “Advanced Practice Nursing”[MeSH Terms]))*	n = 5739
EMBASE (Elsevier)	*(‘critical illness’/exp OR ‘critically ill patient’/exp) AND (‘treatment outcome’/exp OR ‘outcome assessment’/exp) AND (‘intensive care nursing’/exp OR ‘intensive care unit’/exp OR ‘advanced practice nursing’/exp) filtri ‘critical illness’/dm AND ‘human’/de AND ([adult]/lim OR [aged]/lim)*	n = 2029
SCOPUS	TITLE-ABS-KEY ("nurse sensitive outcome*" OR "nursing sensitive outcome*" OR "nurse-sensitive patient outcome*" OR "nursing outcome*" OR "nursing care outcome*") AND ("intensive care" OR "critical care" OR "intensive care unit*" OR "critical care unit*") AND ("adult*" OR "critically ill adult*")	n = 823

Legend. The Table summarises the comprehensive search strategy used in the preliminary scoping review conducted prior to the Delphi rounds. It reports the databases consulted, the specific search strings applied (including keywords, Boolean operators, and filters), and the number of records retrieved from each source. This strategy supported the identification of all studies reporting nursing-sensitive patient outcomes (NSPOs) in adult intensive care settings.
