# Supplementary material for: Development of a Core Set of Nursing-Sensitive Patient Outcomes in Intensive Care Units: A Delphi Consensus Study
Source: Clin Pract. 2026 Apr 30;16(5):89. doi: 10.3390/clinpract16050089 (PMC13206322; doi:10.3390/clinpract16050089)
Supplement: Supplementary file 1 [file clinpract-16-00089-s001.zip › Table S2. Data Summary..pdf]

Table S2. Data Summary.

First author, year	***Country***	***Study design***	***Setting (ICU Type)***	***Participant profile (N)***	***Aim***	***Nursing Intervention/metrics***	***Main outcomes***
**Eggmann et al., 2020**	Switzerland	Randomised controlled trial	Mixed ICU, university hospital.	Mechanically ventilated patients (N = 115).	To evaluate whether early endurance and resistance exercise protocols reduce ICU-acquired weakness and improve functional recovery.	Nurse and physiotherapist delivered early endurance and resistance training.<br>6-Minute Walk Test (6MWT); Functional Independence Measure (FIM); Short Form-36 (SF-36) at six months (m).	Functional outcomes and post-ICU length of stay (LOS) differed significantly by weakness severity: 6MWT 110 m (severe) vs 196 m (moderate) vs 222.5 m (none), *p* = 0.013; FIM *p* = 0.001; LOS after ICU discharge *p* = 0.008. At six months, quality of life and mental health (SF-36) were similar across groups (p = 0.874; p = 0.908).
**Zhou et al., 2021**	USA	Retrospective cohort study	Medical, surgical, and mixed ICUs, large academic center.	Critically adults patients undergoing invasive mechanical ventilation ≥12 h (N = 676).	To determine the incidence, types, risk factors, and clinical consequences of patient–ventilator asynchrony (PVA).	Ventilator waveform analysis to identify types of PVA (double triggering, flow starvation, etc.).<br>In-hospital-free days; duration of mechanical ventilation.	PVA occurred in 24% of ventilation episodes; double triggering (13%) and flow starvation (10%) were most frequent. Pressure-targeted ventilation was associated with reduced PVA (*p* < 0.01). Higher PVA burden, particularly double triggering and flow starvation, was linked to worse outcomes, including prolonged ventilation and fewer hospital-free days (all p < 0.01).
**Launey et al., 2021**	France	Retrospective cohort study	General ICUs, university hospital.	Adults patients (N = 396).	To identify factors associated with delayed defecation after ICU admission and to examine its relationship with ICU length of stay and mortality.	Daily nursing records of bowel function.<br>Time to first defecation after ICU admission; number of bowel movementsstay; mortality.	Delayed defecation was significantly associated with prolonged ICU length of stay, but not with increased mortality.
**Shetty et al., 2018**	Multi-country: China, Japan, Australia	Systematic review of randomised controlled trials (RCTs)	ICUs across multiple countries.	Four RCTs, Adult patients requiring mechanical ventilation (N = 256).	To evaluate whether Bispectral Index (BIS) monitoring improves sedation management compared with clinical evaluation.	BIS monitoring versus clinical evaluation for sedation.<br>Bispectral Index (BIS); Richmond Agitation-Sedation Scale (RASS).	No significant differences were found between BIS and clinical evaluation for ICU length of stay, duration of mechanical ventilation (95% CI: 0.13–0.09), or risk of adverse events.
**Wallace et al., 2012**	USA	Retrospective cohort study	General ICUs.	Adults critically patients (N = 65752).	To evaluate the association between nighttime intensivist staffing and in-hospital mortality.	Daytime staffing intensity (high vs low); nighttime intensivist staffing (presence vs absence).<br>In-hospital mortality.	In ICUs with low-intensity daytime staffing, nighttime intensivists were associated with reduced mortality (aOR 0.62; *p* = 0.04). In ICUs with high-intensity daytime staffing, nighttime coverage did not affect mortality (OR 1.08; *p* = 0.78).

**Dahagam et al., 2011**	USA	Retrospective cohort study	Burn ICUs, university-affiliated center.	Adults patients (N = 462): Diabetic cohort (N = 57); Non-diabetic cohort (N = 405).	To evaluate the effect of diabetic status and glycemic control on clinical outcomes in critically ill burn patients.	Blood glucose levels (admission, mean, variability). Glycemic control via intensive insulin therapy targeting 80–110 mg/dL.	Diabetics were older (60 ± 15 vs 44 ± 17 years), had higher admission glucose (196 ± 81 vs 133 ± 52 mg/dL), and greater glycemic variability (30 ± 11% vs 22 ± 11%). They had fewer ICU-free days (18 ± 12 vs 20 ± 11; *p* < 0.05). Multivariate analysis showed that admission and mean glucose—but not diabetic status—predicted ventilator-free, ICU-free, and hospital-free days. Mortality was independently predicted by age.
**Rose et al., 2012**	Canada	Retrospective cohort study	Prolonged-ventilation weaning centre (PWC).	Adults patients requiring prolonged ventilation admitted for weaning (N = 144).	To evaluate the characteristics and outcomes of patients admitted to a prolonged-ventilation weaning centre.	Weaning success definition: no requirement for positive pressure ventilation (invasive or noninvasive) for ≥7 consecutive days. 24-hour tracheostomy mask trial.	66% achieved a 24-hour tracheostomy mask trial in a median of 15 days. Weaning success was achieved in 53% at a median of 62 days of ventilation,14 days after admission to the PWC. Survival rates post-discharge were 47.3% at 1 year, 34.6% at 2 years, 35.9% at 3 years, and 31.8% at 5 years.
**Underhill et al., 2017**	USA	Retrospective cohort study	General ICUs, academic hospitals.	Adults critically patients who developed line-associated upper extremity deep venous thrombosis (DVT) (N = 193).	To evaluate anticoagulation strategies and clinical outcomes in ICU patients with line-associated upper extremity DVT.	Anticoagulation strategy (therapeutic, prophylactic/subtherapeutic, or none). Clinical assessment of hemorrhage; pulmonary embolism; in-hospital mortality; ICU length of stay.	Major hemorrhage occurred in 15.5% compared with 5.0% for symptomatic pulmonary embolism (*p* < 0.001). In-hospital mortality (34.8% vs 16.7%; *p* = 0.726) and ICU length of stay (33.1 vs 18.3 days; *p* = 0.739) did not differ significantly between patients with major hemorrhage and those with symptomatic pulmonary embolism.
**Wade et al., 2019**	United Kingdom	Cluster-randomised clinical trial	General ICUs.	Critically adult patients (N = 1458).	To evaluate the effectiveness of a nurse-led preventive psychological intervention on post-traumatic stress disorder (PTSD) symptoms in ICU survivors.	Nurse-led preventive psychological intervention: therapeutic ICU environment, three stress-support sessions, relational support versus usual care. PTSD Symptom Scale–Self-Report (PSS-SR).	At 6 months, there was no significant difference in PTSD symptom severity between intervention and control (adjusted difference 0.03; 95% CI − 2.58 to 2.52; *p* = 0.98). No significant effects were observed on anxiety, depression, or health-related quality of life.
**Honiden et al., 2008**	USA	Prospective cohort study	General ICUs, university hospital.	Adults critically patients (N = 178).	To evaluate the effect of early versus late initiation of intravenous insulin therapy on clinical outcomes.	Timing of intravenous insulin therapy initiation ≤ 48 h versus > 48 h after ICU admission. Blood glucose levels mg/dL.	Early initiation was associated with more ventilator-free days (median 12 vs 1; IQR 0–24 vs 0–11), shorter ICU length of stay (6 vs 11 days; IQR 3–11 vs 7–17), and shorter hospital length of stay (15 vs 25 days). Early initiation reduced ICU mortality (OR 0.48) and hospital mortality (OR 0.27; multivariate aOR 0.29).
**Lu et al., 2018**	China	Randomised controlled trial	General ICUs, tertiary hospital	Adult critically patients with expected ICU stay	To evaluate the effectiveness of continuous glucose monitoring systems	Continuous glucose monitoring system (CGMS) versus point-of-care (POC) glucose testing.	CGMS demonstrated superior effectiveness compared with POC testing in optimizing glycemic control among critically ill patients. Specifically, its use was

				≥72 h and admission glucose >10.0 mmol/L (N = 144).	compared with point-of-care glucose measurements in the management of hyperglycemia.	Blood glucose levels (target 8.0–10.0 mmol/L); glucose variability indices; duration of hypoglycemia.	associated with a significantly greater proportion of time spent within the target glucose range (51.5% vs 29.0%; $p < 0.001$) and with reduced glucose variability, as reflected by both the coefficient of variation and standard deviation ($p < 0.05$).
**Rogers et al., 1997**	United Kingdom	Prospective cohort study	General ICU, university hospital	Adults ICU survivors paired with next of kin (N = 99).	To evaluate the reliability of next-of-kin estimates of quality of life (QoL) compared with ICU survivors' self-reports.	Therapeutic relationships and active listening.<br><br>Short Form-36 (SF-36) health survey.	Next-of-kin estimates showed slight-to-fair agreement with patient self-reports, with better concordance for physical than mental domains. Agreement was poor at discharge but improved by 6 months, particularly in physical health dimensions.
**Mehrholz et al., 2015**	Germany	Prospective cohort study	General ICUs, university hospital.	Critically adults patients with ICU-acquired muscle weakness (N = 150).	To evaluate motor recovery and functional outcomes in patients with ICU-acquired muscle weakness undergoing standard rehabilitation.	Functional rehabilitation.<br><br>Functional Status Score for the ICU (FSS-ICU); Functional Ambulation Categories (FAC); Barthel Index (BI).	Median time to walking recovery was 28.5 days (IQR 45) from rehabilitation start and 81.5 days (IQR 64) post-illness onset. FAC predictors of walking recovery included higher FSS-ICU (HR 1.07, 95% CI 1.03–1.12) and functional reach (HR 1.02, 95% CI 1.00–1.04), both $p < 0.05$. Secondary analyses showed significant improvements in ADLs and motor/cognitive function within 8 weeks, except for pain.
**Spada et al., 2011**	USA	Prospective cohort study	Adult general ICUs.	Critically adults patients requiring noninvasive positive pressure ventilation (N = 133)	To evaluate the oxygen saturation/fraction of inspired oxygen (SF) ratio as a predictor of success or failure of noninvasive positive pressure ventilation (NPPV).	Monitoring of arterial blood gases.<br><br>Oxygen saturation/fraction of inspired oxygen (SF) ratio.	NPPV success rate was 41%. Patients without malignancy had higher SF ratios associated with success. An SF ratio < 98.5 predicted NPPV failure with 89% specificity ($p = 0.013$). Among patients requiring ≥24 h of NPPV, a higher tidal volume/predicted body weight ratio negatively correlated with respiratory improvement.
**Tomasi et al., 2012**	Brasil	Prospective cohort study	General ICUs, tertiary hospital.	Adults critically ill patients (N = 162).	To evaluate the association between delirium identified by screening tools and clinical outcomes.	Delirium assessment and management.<br><br>Confusion Assessment Method for the ICU (CAM-ICU); Intensive Care Delirium Screening Checklist (ICDSC), assessed twice daily until ICU discharge or 28 days.	Delirium diagnosed by either tool was associated with longer hospital stay (15.3 vs 10.5 days; $p < 0.001$) and higher mortality. Only CAM-ICU–confirmed delirium was strongly associated with increased mortality (80% vs 5.5%; $p = 0.002$) and prolonged LOS (15.1 vs 10.9 days; $p = 0.04$).
**Peris et al., 2011**	Italy	Prospective cohort study	Trauma ICUs, tertiary hospital.	Major trauma ICU patients (N = 679).	To evaluate the effect of Intra-ICU clinical psychologist support versus usual care on post-traumatic stress (PTSD), anxiety, depression, and quality of life.	Intra-ICU psychological support.<br><br>Hospital Anxiety and Depression Scale (HADS); Impact of Event Scale-Revised (IES-R).	PTSD symptoms (IES-R ≥ 33) were less frequent with psychological support (21.1% vs 57%; $p < 0.0001$). Psychiatric medication use was reduced (8.1% vs 41.7%; $p < 0.0001$). Anxiety (HADS-A > 11: 8.9% vs 17.4%) and depression (HADS-D > 11: 6.5% vs 12.8%) were lower. Patients reported modestly better health-related quality of life ($p = 0.0495$).

**Fernández et al., 2019**	USA	Case study	Surgical, trauma ICU, tertiary hospital.	Critically ill female ICU patients with large, complex wounds (N = 2).	To evaluate the clinical use of negative pressure wound therapy with instillation and dwell time (NPWTi-d).	Application and monitoring of NPWTi-d, wound care support, and preparation for surgical management.<br>Direct clinical wound evaluation (granulation tissue formation, graft take).	Patient 1: After ~2 weeks of NPWTi-d, abundant granulation tissue developed; therapy was switched to conventional NPWT, and definitive surgery was planned after stabilization. Patient 2: After 68 days of NPWTi-d, and NPWT, split-thickness skin grafting achieved 95% graft take, with discharge to rehabilitation 27 days later.
**Happ et al., 2007**	USA	Ethnographic qualitative study	Medical ICU and step-down unit, tertiary center.	Adults critically ill patients undergoing prolonged mechanical ventilation (N = 30); Family members (N = 31); Clinicians (N = 31).	To evaluate care and communication processes during weaning from prolonged mechanical ventilation, with particular attention to the role of patients, families, and clinicians.	Facilitation of patient–family–clinician communication and support during the weaning process.<br>Ethnographic observation and qualitative field notes.	Family members played a significant role in surveillance and observation during the weaning process, influencing care delivery and decision-making. The study emphasized the importance of healthcare providers, including nurses, in recognizing and integrating family roles to enhance care quality and outcomes.
**Payen et al., 2009**	France	Prospective cohort study	General ICUs.	Mechanically ventilated ICU patients (N = 1144).	To evaluate the effect of routine pain assessment on outcomes.	Routine pain assessment on day 2 versus no assessment.<br>Behavioral Pain Scale (BPS), Harris scale, Visual Analog Scale (VAS), Verbal Descriptor Scale (VDS), Numeric Rating Scale (NRS).	Routine pain assessment was associated with shorter mechanical ventilation (8 vs 11 days), shorter ICU length of stay (13 vs 18 days), higher likelihood of ventilator weaning (OR = 1.40), and greater odds of ICU discharge (OR = 1.43).
**Pang et al., 2019**	China	Randomised controlled trial	Neurologic ICUs, tertiary hospital.	Adults patients with cerebral hemorrhage or traumatic brain injury (N = 42): Intervention group (N = 21); Control group (N = 21).	To evaluate the effect of early comprehensive rehabilitation on clinical outcomes.	Early comprehensive rehabilitation program (individualized therapy, neuromuscular facilitation) versus standard monitoring.<br>APACHE II, Medication Regimen Complexity (MRC) score.	The intervention group showed significant improvements in APACHE II and MRC scores, faster consciousness recovery, shorter duration of mechanical ventilation, reduced ICU and hospital length of stay, and fewer complications compared to controls (all $p < 0.05$).
**Obst et al., 2019**	USA	Case series	Surgical, trauma ICUs, tertiary hospital.	Adults patients with complex wounds (N = 6).	To evaluate the application of negative pressure wound therapy with instillation and dwell time (NPWTi-d) for complex wounds.	Application and monitoring of NPWTi-d with reticulated open cell foam (ROCF) dressings, instillation solutions (saline/hypochlorite), and negative pressure −100 to −125 mmHg.<br>Direct clinical wound evaluation.	All patients developed progressive granulation tissue without complications. Wounds were not followed through to closure or grafting.

**Balas et al., 2014**	USA	Prospective, before–after cohort	Medical and surgical ICUs, tertiary hospital.	Adults critically patients (N = 296): Before intervention (N = 146); After intervention (N = 150).	To evaluate the effect of implementing the ABCDE bundle on clinical outcomes.	Implementation of the ABCDE bundle (daily awakening/breathing coordination trials, delirium monitoring/management, early mobilization).<br>Ventilator-free days; Confusion Assessment Method-Intensive Care Unit (CAM-ICU).	Ventilator-free days increased from 21 to 24 (median, *p* = 0.04). Delirium was reduced by 45% (OR 0.55; 95% CI 0.33–0.93; *p* = 0.03). Mobilization out of bed improved from 48% to 66% (OR 2.11; 95% CI 1.29–3.45; *p* = 0.003). No rise in adverse events (self-extubation/reintu-bation). Hospital mortality at 28 days decreased nonsignificantly from 19.9% to 11.3% (OR 0.56; 95% CI 0.28–1.10; *p* = 0.09).
**Ko et al., 2013**	Taiwan	Retrospective cohort study	General ICUs, tertiary hospital.	Mechanically ventilated patients (N = 1453): ICU-acquired bacteremia cohort (N = 126); Non-bacteremia cohort (N = 1327).	To evaluate the impact of ICU-acquired bacteremia (IABs) on clinical outcomes.	Monitoring and management of IABs (≥ 48 h post-admission).<br>Ventilator-free days; ventilator dependence; ICU length of stay, weaning success, ICU mortality.	IABs was associated with prolonged ICU stay (44.8 ± 26.3 vs 21.3 ± 17.8 days; *p* < 0.001), longer ventilator use (55.5 ± 44.8 vs 31.2 ± 25.5 days; *p* < 0.001), lower weaning success (37.3% vs 79.8%; *p* < 0.001), higher ventilator dependence (7.1% vs 1.9%; *p* = 0.032), and increased ICU mortality (48.4% vs 12.7%; *p* < 0.001).
**Tohol et al., 2023**	Palestine	Cross-sectional study	General ICUs.	ICU nurses (N = 215).	To evaluate the use of non-pharmacological pain management methods and perceived barriers among ICU nurses.	Use of non-pharmacological pain management methods.<br>4-point Likert scale assessing frequency of use (16 items) and perceived barriers (6 items).	Over two-thirds of nurses reported using non-pharmacological pain methods; 60% reported high use and 10.2% very high use. Commonly used methods included positioning patients for comfort and communication with patients/families. Main barriers were lack of time/workload (83.7%) and patient instability (77.2%).
**Akroute et al., 2022**	Norway	Phenomenological hermeneutic qualitative study	Adult ICUs, tertiary hospital.	ICU nurses with ≥ 5 years of experience (N = 6).	To evaluate ICU nurses' lived experiences of caring for adult patients with a tracheostomy.	Provision of tracheostomy care.<br>Semi-structured in-depth interviews analyzed using phenomenological hermeneutic thematic content analysis.	Two themes emerged:<br>(1) challenges of caring for tracheostomy patients (communication barriers, ethical dilemmas, need for patience);<br>(2) Satisfaction from providing care (professional growth, intrinsic motivation, and fulfillment).
**Avgerinou et al., 2022**	Greece	Cross-sectional study	General adult ICUs.	ICU nurses (N = 107).	To evaluate ICU nurses' knowledge, practices, and attitudes regarding pressure ulcers (PUs) prevention and management.	Implementation of PUs prevention and management.<br>Sparta Tool PU2014 scale (knowledge 31 items, practices 11 items, attitudes 13 items).	Work experience was not correlated with higher knowledge scores, whereas postgraduate education was positively associated with greater knowledge (*p* = 0.020). Self-assessed knowledge of pressure ulcer causes was moderate. Knowledge of prevention was significantly lower than knowledge of treatment (*p* = 0.001).
**Sweity et al., 2022**	Palestine	Cross-sectional study	General adult ICUs.	ICU nurses (N = 191)	To evaluate ICU nurses' knowledge, attitudes, and practices regarding pain management in critically ill patients.	Implementation of pain management in critically ill patients.	Nurses demonstrated limited knowledge, inconsistent practices, and moderately positive attitudes toward pain management. Policy and organizational barriers further constrained effective pain management delivery.

						Knowledge, Attitudes; Practices (KAP) questionnaire and perceived barriers assessment.	
**Moser et al., 2022**	USA-based, multicountry	Systematic review and meta-analysis	Mixed ICUs, multiple countries.	Adults patients included across 12 studies (N = 9763).	To evaluate prevention strategies for endotracheal and nasotracheal tube–related pressure injuries.	Implementation of pressure injury prevention for endotracheal and nasotracheal tubes (anchor fastening devices, serial assessment/repositioning, nasal barrier dressings).<br>Incidence of device-related pressure injuries.	Endotracheal tube pressure injury incidence was reduced from 4.2% (control) to 1.0% (intervention); barrier device reduced risk (RR 0.54; 95% CI 0.38–0.77; *p* = .001). Nasotracheal tube injury incidence decreased from 21.1% to 10% with nasal alar dressings (53% absolute reduction). Serial assessment/repositioning showed no conclusive pooled benefit.
**Zelalem et al., 2024**	Ethiopia	Cross-sectional study	General adults ICUs.	ICU healthcare operators (N = 237).	To evaluate healthcare professionals' knowledge regarding detection of patient–ventilator asynchrony (PVA).	Implementation of PVA detection using waveform analysis.<br>Structured interviewer-administered questionnaire assessing knowledge.	Attendance at ≥ 3 mechanical ventilation training sessions was strongly associated with higher knowledge of PVA detection (aOR 6.88; 95% CI 2.61–15.45). Offsite mechanical ventilation training was also linked to better knowledge compared to onsite training (aOR 2.63; 95% CI 1.36–7.98).
**Torres et al., 2021**	Multi-country: Brasil, Peru, Argentina, Chile, Paraguay, Uruguay	Cross-sectional study	General adult ICUs.	Expert ICU nurses (N = 40).	To evaluate the validity of a nursing care instrument designed to prevent unplanned extubation (UPE).	Implementation of nursing care instrument for UPE prevention.<br>Content Validity Index (CVI).	The instrument demonstrated high overall content validity (CVI = 0.91). Two interventions scored below the accepted CVI threshold (< 0.78). Universal agreement was lower among Brasilian nurses (4.0%) compared to foreign nurses (26.6%). Fleiss' κ values varied (–0.067 to 0.067) across components, indicating limited inter-rater reliability.
**Gobeil et al., 2023**	Canada	Retrospective before–after cohort	Adult ICUs, tertiary hospital.	Adults critically patients (N = 400):<br>Before intervention (N = 200);<br>After intervention (N = 200).	To evaluate the impact of installing virtual windows in windowless ICU rooms on the incidence of delirium.	Implementation of virtual windows (paintings depicting outdoor scenes) in windowless ICU rooms.<br>Intensive Care Delirium Screening Checklist (ICDSC).	After adjustment for confounding factors, no statistically significant difference in delirium incidence was observed between groups (OR 0.906; 95% CI 0.584–1.402; *p* = 0.656).
**Rebouças et al., 2020**	Brasil	Cross-sectional study	General adult ICUs, tertiary hospital.	ICU nurses (N = 11).	To evaluate ICU nurses' practices related to the prevention of pressure injuries.	Implementation of pressure injury prevention practices.<br>Positivity Index (PIx) to classify quality of care.	The general average PIx was 57.8% (± 13.8), indicating poor and unsafe nursing practice regarding pressure injury prevention.

**Eberle et al., 2019**	Brasil	Descriptive exploratory qualitative study	General adult ICUs, tertiary hospital.	ICU nurses (N = 6).	To evaluate ICU nurses' perspectives on non-pharmacological strategies for delirium management.	Implementation of non-pharmacological delirium management strategies.<br><br>Semi-structured interviews.	Nurses reported awareness of delirium and identified several non-pharmacological strategies for its prevention and management, including verbal orientation, use of clocks/calendars, presence of family members, and minimizing environmental stressors.
**Rivera-Romero et al., 2019**	Colombia	Hermeneutic phenomenological qualitative study	General adult ICUs, tertiary hospital.	ICU nurses (N = 18).	To evaluate ICU nurses' experiences in providing care to families of patients during end-of-life situations.	Implementation of family support during end-of-life care.<br><br>Semi-structured interviews analyzed with thematic content analysis.	Nurses reported significant emotional and psychological burdens when supporting families during end-of-life care. A lack of specific training in end-of-life and family support was identified. In the absence of formal preparation, nurses relied on personal experiences and empirical practices.
**Sinfronio et al., 2023**	Brasil	Retrospective cohort study	General adult ICUs, tertiary hospital.	Adults patients (N = 1233).	To evaluate the incidence, causes, and outcomes of ICU readmissions.	Monitoring and management of ICU readmissions.<br><br>Retrospective chart review.	Readmission rate was 4.7%. Mean ICU length of stay was 10.6 days and hospital stay was 35.8 days. Primary causes of readmission were respiratory complications (27.6%) and sepsis (24.1%). Mean time to readmission was 5.6 days. Mortality among readmitted patients was 70.7%.
**Monnerat et al., 2023**	Brasil	Prospective cohort study	General adult ICUs, tertiary hospital.	Adults patients undergoing suctioning events (N = 25).	To evaluate adherence to best practices during endotracheal suctioning.	Implementation of endotracheal suctioning procedure.<br><br>Observational checklist of procedural steps.	Nursing staff performed 52% of suction events, physiotherapists 44%, and physicians 4%. Oxygen fraction was not adjusted before/after in 80% of cases. Tube clamping during insertion was omitted in 60% of relevant cases. Adherence to protective equipment was high (mask 100%, gown 92%, gloves 100%).
**Ataeeara et al., 2024**	Iran	Randomised clinical trial	General ICUs, tertiary hospital	Adults patients and family members (N = 200): Intervention group (N = 100); Control group (N = 100).	To evaluate the effectiveness of a transition nursing program designed to facilitate transfer from ICU to general units, including pre-transfer education, accompaniment, and post-transfer follow-up.	Implementation of transition nursing program (pre-transfer education, accompaniment during transfer, post-transfer follow-up by liaison nurses).<br><br>Spielberger State-Trait Anxiety Inventory (STAI), researcher-developed satisfaction questionnaire.	Patients in the intervention group had significantly lower post-transfer state anxiety (Mean ± SD: 34.68 ± 8.02) compared to controls (53.50 ± 10.91; $p < 0.0001$). Family members also reported reduced anxiety (32.52 ± 7.84 vs 52.12 ± 10.21; $p < 0.0001$). Satisfaction levels were higher in the intervention group.
**Bilodeau et al., 2018**	Canada	Cross-sectional study	General adult ICUs, multiple hospitals.	ICU nurses (N = 237).	To evaluate ICU nurses' practices related to the early mobilization of mechanically ventilated patients.	Implementation of early mobilization practices in mechanically ventilated patients.<br><br>Electronic survey.	Barriers to implementation included patient instability, lack of resources, and insufficient training. Facilitators included interdisciplinary collaboration and established protocols. Findings emphasize the need to address barriers to strengthen early mobilization practices and improve patient outcomes.

**Nezhad et al., 2023**	Iran	Quasi-experimental study	General ICUs, tertiary hospital.	Adults critically patients (N = 40).	To evaluate the effects of Swedish massage versus earplugs and eye masks on sleep quality in ICU.	Implementation of non-pharmacological sleep improvement strategies.<br>Sleep quality measurement tools.	Both interventions significantly improved sleep quality scores ($p = 0.001$). Swedish massage yielded higher mean post-intervention sleep quality (202 ± 46.03) compared to earplugs/eye masks (184.79 ± 39.5; $p = 0.037$).
**Orinovsky et al., 2018**	Israel	Retrospective cohort before–after study	General ICUs, tertiary hospital.	Adults patients (N = 117):<br>Before intervention (N = 65);<br>After intervention (N = 52).	To evaluate the impact of a nurse-led, evidence-based enteral nutrition (EN) protocol on clinical nutrition outcomes.	Implementation of a nurse-led, evidence-based EN feeding protocol.<br>Body Mass Index (BMI), time to initiation of EN (hours).	Time to EN initiation was reduced in the intervention group (52.3 ± 42.6 h vs 70.3 ± 65.2 h; $p = 0.007$). Achievement of caloric targets within 96 h was higher (90% vs 34%; $p < 0.001$). Feeding intolerance was lower (6% vs 14%; $p = 0.03$).
**Lei et al., 2023**	China	Randomised controlled trial	General ICUs, tertiary hospital	Mechanically ventilated adults patients (N = 90):<br>intervention group (N = 45);<br>control group (N = 45).	To evaluate the effectiveness of a structured oral care protocol on oral health and Ventilator-Associated Pneumonia (VAP).	Implementation of a structured oral care protocol.<br>Oral Health Assessment Tool (OHAT); VAP incidence; duration of mechanical ventilation.	Oral health improved significantly in the intervention group (OHAT mean 1.89 ± 0.34 vs 4.26 ± 0.51; $p < 0.05$). The intervention group also showed reduced incidence of VAP, shorter ICU stay, and decreased duration of mechanical ventilation compared with controls.
**Oshvandi et al., 2024**	Iran	Clinical trial	General ICUs, tertiary hospital.	Adult Intubated patients (N = 130).	To evaluate the effects of licorice mouthwash versus chlorhexidine mouthwash on oral health.	Implementation of oral care protocol.<br>Daily oral health assessment.	Both licorice and chlorhexidine mouthwashes significantly improved oral health in mechanically ventilated patients after intervention ($p < 0.05$). Licorice mouthwash was as effective as chlorhexidine in enhancing oral health.
**Secombe et al., 2019**	Australia	Retrospective cohort study	General ICUs, tertiary hospital.	Adults patients admitted with primary skin and soft-tissue infections (SSTIs) (N = 80).	To evaluate the incidence, characteristics, and outcomes of ICU admissions for SSTIs.	Monitoring and management of ICU patients with SSTIs.<br>SSTI incidence; associated clinical outcomes.	Annual ICU admission rate for SSTIs was 24.2 per 100,000 population. Pathogen isolates included 50% Gram-positive cocci and 20% multi-resistant organisms. Mortality was 0% at 28 days post-discharge and 1.3% at 90 days.
**Muhammad et al., 2022**	Iraq	Cross-sectional study	Critical Care Units (CCU) and Electronic Surveillance Units (ESU), tertiary hospital.	CCU and ESU nurses (N = 167).	To evaluate the patient safety culture from the perspective of nurses working in CCU.	Implementation of patient safety practices.<br>Hospital Survey on Patient Safety Culture (HSOPSC) questionnaire.	67.7% of nurses rated the patient safety culture as acceptable, whereas 6.6% perceived it as very weak.

**de Sousa Nóbrega et al., 2023**	Brasil	Cross-sectional study	General ICUs, teaching hospital	ICU nurses (N = 22); nursing technicians (N = 14); undergraduate nursing students (N = 34).	To evaluate the knowledge of ICU nurses, nursing technicians, and nursing students regarding pressure ulcer prevention.	Implementation of pressure ulcer prevention practices.<br>Pieper's Pressure Ulcer Knowledge Test (PUKT).	Only 22.7% of nurses, 7.1% of technicians, and no students achieved the ≥ 90% benchmark, revealing significant knowledge gaps. Statistical analyses (ANOVA, Shapiro–Wilk, Levene's) confirmed these deficiencies, underscoring the need for targeted educational programs in ICU settings.
**Karaagac et al., 2023**	Turkey	Cross-sectional study	General ICUs, tertiary hospital.	Alert and conscious adults patients (N = 111).	To evaluate the impact of environmental stressors on comfort levels of ICU patients.	Implementation of environmental stressor management and comfort assessment.<br>ICU Environmental Stressors Scale (ICUESS), General Comfort Scale (GCS), Numeric Pain Scale.	Patients' overall comfort levels were above average (mean GCS = 135.24 ± 18.04). A moderate negative correlation was found between ICUESS and GCS scores (r = –0.57; *p* < 0.001), indicating that higher perceptions of environmental stressors were associated with lower comfort levels.
**Araújo et al., 2023**	Brasil	Cross-sectional study	General ICUs, tertiary hospital.	Adults patients (N = 206)	To evaluate signs and symptoms associated with the nursing diagnosis of ocular dryness.	Implementation of ocular dryness assessment.<br>Custom data collection instrument analysed through latent class modeling (sensitivity, specificity, prevalence of indicators).	Dry eye was highly prevalent (76.2%). Indicators with highest sensitivity included mucoid filaments (100%), excessive mucous secretion (99.9%), vascular congestion on ocular surface (74.5%), and chemosis (71.9%). Tear volume reduction showed strong specificity (81.6%), confirming its diagnostic reliability for ruling out ocular dryness.
**dos Santos et al., 2022**	Brasil	Hermeneutic phenomenological qualitative study	General adult ICUs, tertiary hospital.	ICU nurses (N = 32).	To investigate the challenges faced by ICU nurses in applying the CAM-ICU tool for delirium assessment in mechanically ventilated patients.	Implementation of delirium assessment using the CAM-ICU tool.<br>Semi-structured interviews analysed through content analysis.	Nurses reported knowledge deficits and lack of formal training in delirium assessment, resulting in difficulty distinguishing delirium from other conditions. Challenges also arose from limited patient cooperation due to sedation or illness severity.
**Solimani et al., 2022**	Iran	Clinical trial	General ICUs, tertiary hospital	Adults critically patients (N = 52): Intervention group (N = 26); Control groups (N = 26).	To evaluate the effect of white noise on pain levels in ICU patients.	Implementation of white noise intervention (30 minutes for 3 consecutive days).<br>Behavioral Pain Scale (BPS).	Mean baseline BPS scores were comparable between groups (4.98 ± 0.84 vs 5.15 ± 1.09). After intervention, the white noise group had significantly lower pain scores (4.16 ± 0.74) compared to controls (4.97 ± 1.08; *p* < 0.001).
**Acun et al., 2022**	Turkey	Prospective cohort study	General ICUs, tertiary hospital	Mechanically ventilated adults patients (N = 38); ICU nurses involved in bundle compliance training	To evaluate the impact of a ventilator-associated event (VAE) prevention bundle on infection incidence and compliance rates.	Implementation of VAE prevention bundle (head of bed elevation, oral chlorhexidine rinse, aseptic aspiration, ventilator circuit maintenance).	Bundle compliance reached 85%. Infection rates decreased across pre-, during, and post-intervention periods. Despite an increased number of patients, ventilator days decreased in the post-intervention period, indicating improved outcomes.

				(N = 8).		VAE incidence; bundle compliance percentage.	
**Niazi et al., 2022**	Iran	Randomised controlled trial	General ICUs, tertiary hospital.	Adults patients (N = 60): Intervention group (N = 30); Control group (N = 30).	To evaluate the effects of pulmonary rehabilitation, including inspiratory muscle training and psychological interventions, on clinical and psychological outcomes.	Implementation of pulmonary rehabilitation program (inspiratory muscle training and psychological interventions).<br>Inspiratory muscle strength index (Sindex); standardised psychological questionnaires.	Inspiratory muscle strength improved significantly in the intervention group ($p < 0.005$). ICU length of stay was reduced compared to controls. Psychological outcomes (post-operative stress, depression, and social pain) were also significantly improved in the intervention group.
**Gungor et al., 2021**	Turkey	Cross-sectional study	General ICUs, tertiary hospital.	Adults patients (N = 77).	To evaluate the impact of environmental noise on patients' vital signs and anxiety levels in the ICU.	Implementation of environmental noise monitoring and patient assessment. Vital signs monitoring (blood pressure, pulse, respiratory rate, temperature), State-Trait Anxiety Inventory (STAI), Visual Analogue Scale (VAS), sound level meter (dB).	Mean ICU noise level was 56.18 dB, exceeding the WHO recommended maximum of 40 dB. No significant correlation was found between noise levels and patients' vital signs ($p > 0.05$). No significant relationship was observed between noise levels and state or trait anxiety scores ($p > 0.05$).
**Nuwi et al., 2018**	Indonesia	Systematic review of randomized controlled trials	General ICUs, multi-country.	Adult critacally patients with ICU stay > 24 h and Mechanical ventilation ≥ 48 h (N = 6 studies).	To evaluate the effects of active mobilization interventions on physical function, quality of life, and clinical outcomes in critically ill ICU patients.	Implementation of active mobilization interventions.<br>Functional Status Score, Medical Research Council (MRC) Score, Functional Independence Measure (FIM), 6-Minute Walk Test (6MWT).	Active mobilization improved physical function, mobility, and functional status. Health-related quality of life was enhanced post-discharge. Some studies reported reduced ICU and hospital length of stay, though results were inconsistent. Mechanical ventilation duration was variably reduced.
**Khasal et al., 2022**	Iraq	Cross-sectional study	General adult ICUs, tertiary hospital.	ICU nurses with ≥ 1 year of experience (N = 50).	To evaluate ICU nurses' knowledge and practices regarding the prevention of catheter-associated urinary tract infections (CAUTIs).	Implementation of CAUTI prevention practices.<br>Knowledge and practices questionnaire for CAUTI.	54% of nurses demonstrated fair knowledge. Preventive measures most often identified included antimicrobial prophylaxis and early catheter removal (72%) and antiseptic cleansing (70%). Knowledge level was not significantly associated with demographic characteristics, except for training courses attended.
**Ketcham et al., 2022**	USA	Retrospective cohort before–after study	General ICUs, tertiary hospital.	Adults patients (N = 56): Before-intervention (N = 29); After-intervention (N = 27).	To evaluate the impact of a nurse-driven spontaneous awakening trial (SAT) protocol on sedation use, ventilation duration, and mortality.	Implementation of a nurse-driven SAT protocol.<br>SATs performed per eligible days; duration of sedative infusion; duration of mechanical ventilation; in-hospital mortality.	SAT performance improved significantly post-intervention (ratio 0.91 vs 0.52; $p < 0.01$). Median sedative infusion duration decreased (2.3 vs 3.6 days; $p = 0.02$). Ventilation duration was reduced but not statistically significant (3.8 vs 4.7 days; $p = 0.18$). In-hospital mortality remained unchanged (41%).
**Domingues et al., 2022**	Brasil	Cross-sectional study	General ICUs, tertiary hospital	Adults patients (N = 105)	To evaluate the prevalence of incontinence-associated	Implementation of IAD surveillance through medical	IAD prevalence was 9.5% (10/105 patients). Admission due to trauma was significantly associated with IAD occurrence ($p = 0.02$).

					dermatitis (IAD) and its associated factors.	record review and physical examination.<br><br>Prevalence of IAD.	
**dos Reis et al., 2022**	Brasil	Observational study	General ICUs, tertiary hospital.	Adults patients (N = 122).	To evaluate functional status of ICU patients after admission.	Administration of the scoring tool and collection for the degree of independence and autonomy of a person in performing basic activities of daily living.<br><br>Barthel Index.	The Barthel Index showed moderate to high correlations with other physical functioning measurement instruments (Spearman's rho = 0.57 to 0.88; $p < 0.001$ for all), supporting its construct validity.
**Oliveira et al., 2019**	Portugal	Quasi-experimental pre–post study	General ICUs, tertiary hospital.	Adults patients (N = 122):<br>Intervention group (N = 61);<br>Control group (N = 61).	To evaluate the impact of a ventilatory weaning protocol on weaning duration and adverse events in ICU patients.	Implementation of a ventilatory weaning protocol, including staff training and standardized weaning log sheet.<br><br>Weaning duration (days); incidence of adverse events.	Weaning duration was shorter in the intervention group (mean 2.1 vs 3.31 days; 36.6% reduction; $p = 0.048$). No significant differences were found in adverse events.
**Sánchez Peña et al., 2021**	Colombia	Quasi-experimental pre–post study	General ICUs, tertiary hospital.	Mechanically ventilated adults patients (N = 171):<br>Pre-intervention (N = 101);<br>Post-intervention (N = 70).	To evaluate the effect of an educational programme for nursing staff on oral care practices and ventilator-associated pneumonia (VAP).	Implementation of an educational programme on oral hygiene techniques tailored to patient condition.<br><br>Incidence of VAP, duration of mechanical ventilation (days).	Daily oral care provision increased significantly post-intervention (29.6% vs 92.8%; $p < 0.001$). VAP incidence decreased from 8.9% to 2.8% (incidence rate ratio reduction from 9 to 3.5 per 1,000 ventilator-days; $p = 0.488$).
**Jansen et al., 2020**	Brasil	Cross-sectional study	General ICUs, tertiary hospital.	Adults patients (N = 67).	To evaluate the risk of developing pressure ulcers (PUs) among ICU patients.	Implementation of skin integrity surveillance.<br><br>Braden Scale.	Among patients classified at severe risk on the Braden Scale, 83.3% developed PUs, while 76.7% did not. Patients who developed PUs had significantly longer ICU stays (8 vs 3 days; $p < 0.001$).
**Pessoa et al., 2019**	Brasil	Integrative literature review	General ICUs, elderly focus	Articles focusing on delirium in elderly adults patients (N = 6).	To evaluate nursing interventions for the prevention, detection, and management of delirium in elderly ICU patients.	Implementation of nursing interventions for delirium prevention and management.<br><br>Confusion Assessment Method for the ICU (CAM-ICU).	Nursing interventions emphasized early recognition and non-pharmacological strategies (environmental modifications, cognitive stimulation). Educational programs for nurses were highlighted as essential to improve knowledge and skills in delirium management.
**Amundadottir et al., 2019**	Iceland	Randomised controlled trial	General ICUs, tertiary hospital	Adults patients (N = 50):<br>Intervention group (N = 29);<br>Control group (N = 21).	To evaluate the effect of increased frequency of vertical mobilisation on physical function and recovery in ICU patients.	Implementation of vertical mobilisation twice daily versus once daily.<br><br>SF-36v2, 6-Minute Walk Test (6MWT), Medical Research Council Sum Score (MRC), Modified Barthel Index.	Mobilisation occurred on 31% of ICU days in the intensive group versus 22% in controls ($p = 0.03$). No significant differences were observed between groups in duration of mechanical ventilation, ICU/hospital length of stay, health-related quality of life, or physical function at 12-month follow-up.

**Wei et al., 2022**	China	Cross-sectional study	Surgical ICUs, tertiary hospital.	ICU nurses and head nurses (N = 173).	To examine oral care practices of ICU nurses for post-operative oral cancer patients.	Implementation of oral care practices.<br>Structured online questionnaire.	Routine oral hygiene was performed by 91.2% of nurses at fixed times, most commonly using swabbing (91.9%), saline mouthwash (82.7%), and cotton ball tools (86.1%). Barriers included lack of knowledge/skills (76.9%) and absence of training (69.4%). Almost all nurses (98.8%) expressed a need for standardized training.
**Grimm et al., 2020**	USA	Narrative literature review	General adult ICUs.	Articles focusing on sleep in ICU patients (N = 54).	To identify evidence-based approaches to address sleep deprivation.	Implementation of nursing interventions to address sleep disruption.<br>Richards-Campbell Sleep Questionnaire (RCSQ).	Sleep deprivation was identified as a significant contributor to delirium in ICU patients. Modifiable risk factors included frequent care interactions, light exposure, noise, and medication effects.
**Kavaklı et al., 2023**	Turkey	Randomised controlled trial	General ICUs, tertiary hospital	Adults patients (N = 100): Intervention group (N= 50); Control group (N = 50).	To evaluate the effect of eye masks and anxiety-reduction counselling on sleep quality and anxiety.	Application of eye masks during nighttime sleep combined with therapeutic communication and patient education.<br>Richards-Campbell Sleep Questionnaire (RCSQ); Hamilton Anxiety Rating Scale (HARS).	Sleep quality improved in the intervention group (RCSQ 316.40 ± 148.42 vs 291.80 ± 149.29; p = 0.411). Anxiety levels were significantly reduced in the intervention group (HARS 11.44 ± 8.74 vs 15.38 ± 10.49; p = 0.044). A moderate negative correlation between sleep quality and anxiety was found (r = –0.395; p = 0.005).
**Hasanin et al., 2017**	Egypt	Prospective cohort study	General ICUs, tertiary hospital.	Sedated, non-intubated adults patients (N = 87).	To evaluate the perfusion index (PI) as a tool for pain assessment.	Implementation of pain assessment through perfusion index measurement.<br>Perfusion Index (PI); Behavioral Pain Scale for Non-Intubated patients (BPS-NI); Richmond Agitation-Sedation Scale (RASS).	No significant correlations were found between absolute PI values and ABP, BPS-NI, or RASS at baseline or after stimulation. However, a strong negative correlation was observed between PI changes and BPS-NI changes (r = –0.616; p < 0.001).
**Leigher et al., 2020**	USA	Prospective before–after cohort study	General ICUs, tertiary hospital.	Adults patients (N = 62): Before-intervention (N = 32); After-intervention (N = 30).	To evaluate the effect of chest skin washing and ECG electrode replacement on monitor alarm frequency.	Implementation of chest skin washing with soap and water plus replacement of ECG electrodes at shift start.<br>Monitor alarm frequency; hygiene status.	Clinical alarms per 8 hour shift decreased significantly in the post-intervention cohort compared to the pre-intervention cohort, indicating improved monitoring reliability and reduced alarm burden.

**Ramirez et al., 2020**	USA	Retrospective cohort study	General ICUs, tertiary hospital.	Mechanically ventilated adults patients (N = 30).	To evaluate the implementation of an oxygen-weaning protocol aimed at reducing hyperoxia.	Monitoring of parameters useful for the implementation of the weaning protocol with accelerated titration of oxygen therapy.<br><br>Parameters: time to first reduction of FiO_2 after fulfilling the weaning criteria, percentage change of FiO_2, arterial blood gases (PaO_2, SaO_2).	The most common SpO_2 threshold for initiating oxygen weaning was 92%. Mean FiO_2 before extubation was 39.6% ± 15.3%. Adequate oxygenation was observed with mean PaO_2 of 99.3 ± 6.7 mmHg and SaO_2 of 95.1% ± 26.9%. Mean time to first FiO_2 adjustment post-criteria was 9.1 ± 10.6 h (range 1–39; IQR 2–13).
**Alıcı et al., 2020**	Turkey	Cross-sectional study	General ICUs, tertiary hospital.	Adults patients (N = 317).	To evaluate the effects of quality of life and life satisfaction on perceptions of individualized nursing care.	Assessment of individualized nursing care perceptions.<br><br>Short Form-36 (SF-36) Quality of Life Scale, Satisfaction with Life Scale; Individualized Care Scale – Patient Version A & B (ICS-A, ICS-B).	Patients reported perceptions of individualized nursing care above moderate levels. Significant associations were observed between quality of life, life satisfaction, and individualized care perceptions. ICS-A (3.5 ± 0.7), ICS-B (3.3 ± 0.6), SF-36 and Satisfaction with Life Scale (21.7 ± 5.9) scores varied according to sociodemographic and clinical characteristics.
**Hayakawa et al., 2020**	Brasil	Clinical trial	General ICUs.	Adults patients, right eyes intervention group and left eyes control group (N = 27).	To evaluate the effectiveness of transparent polyurethane film in preventing ocular surface injuries.	Bilateral eye care with 0.9% saline and the right eye was occluded with a transparent polyurethane film versus the left eye remained uncovered, serving as a control.<br><br>Incidence of ocular surface injuries.	Occlusion with polyurethane film significantly reduced ocular surface injuries compared to uncovered eyes: right eye mean = 4.1 days without injury vs left eye mean = 2.4 days without injury ($p = 0.002$).
**Ceron et al., 2020**	USA	Retrospective cohort study	General adult ICUs.	Tracheostomised adults patients (N = 18).	To evaluate the impact of speaking valve use during the weaning process on mobility outcomes.	Implementation of nurse-led facilitation of speaking valve use during ventilator weaning.<br><br>Perme Intensive Care Unit Mobility Score.	Perme scores increased from a median of 11.3 (IQR 10.1–12.0) pre-intervention to 18.2 (IQR 16.2–20.1) immediately after initiation ($p = 0.01$). Mobility improveme.
**da Costa et al., 2019**	Brasil	Prospective cohort study	Obstetric ICU.	High-risk pregnant women (N = 607).	To identify the incidence and characteristics of adverse drug reactions (ADRs).	Nurse-led daily active pharmacovigilance for ADR detection.<br><br>Naranjo's algorithm.	Incidence of ADRs was 27.2%, with 29.7% moderate reactions and no severe cases. Magnesium sulfate was the most frequent drug involved (25.2%), with 44.5% of exposed patients developing ADRs: drowsiness (68.6%), absent patellar reflex (21.6%), hypotension (9.8%).

**Charissa et al., 2019**	Australia	Systematic review	Multi-ICU.	Mechanically ventilated patients with endotracheal tube and tracheostomised patients<br>(N = 48 studies)	To evaluate the feasibility, usefulness, and safety of augmentative and alternative communication (AAC) or tracheostomy-related interventions.	Nurse-facilitated use of augmentative and alternative communication (AAC) methods and tracheostomy-related interventions; metric: communication success rates with AAC.	47 of 48 studies confirmed feasibility of AAC interventions. Sixteen studies assessed safety, reporting no serious adverse events. Speaking valve tolerance ranged from 74% to 100%. Communication success rates with AAC boards were 80–90%, with time to communication after intervention ranging 1–4 days.
**Ahtiala et al., 2018**	Finland	Retrospective cohort study	General ICUs.	Adults critically patients<br>(N = 3196)	To evaluate the risk of pressure ulcers (PUs) among ICU patients with high care dependency.	Nurse-led pressure ulcer risk monitoring.<br>Braden Scale and incidence of PUs.	Overall PUs incidence was 8.7%. ICU stay > 3 days was associated with a 24.5% incidence compared to 2.4% for stays < 3 days ($p = 0.001$). Low hemoglobin (< 100 g/L) significantly increased PU risk ($p = 0.001$).
**Çiftçi et al., 2015**	Turkey	Clinical trial	Neuro-ICU.	Cerebrovascular adul patients<br>(N = 72).	To evaluate the effects of Turkish classical music therapy on pain, anxiety, comfort, and vital signs in ICU patients with cerebrovascular disease.	Nurse-delivered music therapy intervention.<br>Visual Analogue Scale (VAS); Faces Anxiety Scale; State-Trait Anxiety Inventory (STAI-1); General Comfort Questionnaire.	Pain scores (VAS) decreased significantly from 2.3 ± 1.7 to 0.7 ± 1.2 ($p < 0.05$). Anxiety scores (Faces Anxiety Scale) decreased from 3.9 ± 2.0 to 1.2 ± 1.5 ($p < 0.05$). State anxiety (STAI-1) decreased from 41.5 ± 10.4 to 34.7 ± 8.6 ($p < 0.05$). Comfort scores increased from 135.3 ± 11.8 to 142.7 ± 11.0 ($p < 0.05$).
**Garcia et al., 2019**	Brasil	Cross-sectional study	General ICUs, multi-hospitals.	Adults patients<br>(N = 11).	To evaluate the relationship between nursing care time per patient/day and the incidence of device-related adverse events.	Nurse-provided care time for ICU patients.<br>Incidence of device-related adverse events (oro/nasogastroenteric probe, endotracheal tube, central venous catheter).	Overall, the incidence of device loss was: oro/nasogastric tube 2.19/100 patient days; unplanned extubation 0.42/100 patient days; central venous catheter 0.22/100 patient days. No significant correlation was observed between nursing hours and the incidence of adverse events.
**Garcia et al., 2012**	Brasil	Descriptive-correlational observational study	General ICUs, multi-hospitals	Adults patient studies<br>(N = 11).	To evaluate the association between nursing care time and quality of care indicators related to device management.	Nurse care hours per patient/day.<br>Incidence of unplanned feeding tube exit, unplanned extubation, and central venous catheter (CVC) loss.	Longer nursing care time was associated with reduced unplanned extubation rates. No significant correlation was found between nursing care time and outcomes related to feeding tube or central venous catheter loss.
**Yazdannik et al., 2019**	Iran	Randomised, crossover clinical trial	General ICUs.	Mechanically ventilated adults patients<br>(N = 50).	To evaluate the efficacy of closed suction systems at two different suction pressures.	Closed Suction System (CSS) applied at 100 mmHg vs 200 mmHg.<br>Secretion clearance efficacy (absence of secretion flow after 10 seconds).	Closed suction at 200 mmHg was significantly more effective in secretion removal compared to 100 mmHg (95% CI: 1.4–2.0; $p = 0.0001$). No additional safety concerns were observed, supporting its clinical use.
**de Araujo et al., 2019**	Brasil	Randomised controlled trial	General ICUs.	Mechanically ventilated adults patients	To evaluate the effectiveness of artificial tear	Nursing eye care intervention: liquid artificial tears (Lacribell)	Artificial-tear gel significantly reduced dry-eye incidence (9% vs 21%; RR = 0.40; 95% CI: 0.166–0.964; $p = 0.04$). Incidence per 100 patient-days was

				(N = 140):<br>Intervention group<br>(N = 70);<br>Control group<br>(N = 70).	formulations in preventing dry-eye complications.	versus gel artificial tears (Vidisic Gel) every 4 h for 5 days.<br>Fluorescein eye stain test, Schirmer test.	1.72 vs 4.28, supporting the gel formulation as a superior preventive nursing measure.
**McGee et al., 2019**	USA	Retrospective cohort study	General ICUs.	Adults patients<br>(N = 2,723).	To evaluate the prevalence and clinical impact of pressure ulcers (PUs).	Nursing skin assessment at ICU admission.<br>APACHE III score.	PUs were present in 6.6% of patients at admission. Patients with PUs had longer hospital stays (15.6 vs 10.5 days; *p* < 0.001) and higher in-hospital mortality (32.2% vs 18.3%; *p* < 0.001). After adjustment for APACHE III score, PUs remained associated with prolonged hospital stay (mean increase 3.1 days; 95% CI: 1.5–4.7; *p* = 0.001) but not with mortality.
**Momennasab et al., 2019**	Iran	Observational descriptive study	Cardiothoracic ICU.	Adults patients<br>(N = 35);<br>ICU nurses<br>(N = 10).	To evaluate non-verbal communication skills between patients undergoing mechanical ventilation and intensive care nurses.	Observation of non-verbal communication.<br>Satisfaction assessed with a 6-item Likert scale 8–12 hours post-extubation.	Patients initiated most communications (75%), mainly regarding physical needs (50.3%) and pain (23.5%), predominantly using non-verbal gestures (88.6%). Satisfaction was low: 45% of nurses and 54% of patients rated communication as “very low.” None of the nurses and only 5.7% of patients reported full satisfaction.
**Smith, 2007**	USA	Prospective cohort study	General ICU.	Diabetic patients<br>(N = 212):<br>Computer-guided protocol<br>(N = 88);<br>Paper-based protocol<br>(N = 124).	To evaluate the effectiveness and safety of a nurse-driven computerized insulin infusion protocol compared to a paper-based protocol for blood glucose control.	Insulin therapy management and Blood glucose monitoring.<br>Glucose meter (mg/dL).	Of 11,076 glucose measurements, 56.5% were within 80–110 mg/dL and 79.9% within 80–150 mg/dL. Severe hypoglycemia (<40 mg/dL) occurred in <0.01% of checks (3/11,076). Nurses reported high acceptance, with 89% considering it safe and 93% preferring it over previous methods.
**Taslimi, 2018**	Iran	Quasi-experimental study	General ICU.	Intubated adults patients<br>(N = 60):<br>Intervention group<br>(N = 30);<br>Control group<br>(N = 30).	To evaluate the effectiveness of continuous endotracheal tube (ETT) cuff pressure monitoring compared to intermittent monitoring.	ETT cuff pressure monitoring (continuous with pressure transducer vs intermittent with manual manometer every 8 hours).<br>ETT cuff pressure (cmH_2O).	Continuous monitoring was significantly more effective in maintaining optimal cuff pressure (85% versus 60%; p = 0.05) and in reducing cuff-related complications compared to intermittent monitoring.

**Silva et al., 2017**	Brasil	Cross-sectional study	General adults ICU.	ICU nurses (N = 104).	To evaluate the knowledge of ICU nurses regarding blood transfusion procedures across pre-transfusion, transfusion, and post-transfusion stages.	Blood transfusion management.<br>Self-developed and validated checklist instrument covering pre-transfusion, transfusion, and post-transfusion stages.	Nurses who had received training and guidance scored higher compared to those without (51.9% vs. 41.9%; *p* = 0.002). Higher self-confidence was associated with better knowledge (51.6% vs. 36.4%; *p* < 0.001), and postgraduate education was also positively correlated with knowledge levels (55.3% vs. 48.4%; *p* = 0.005).
**Manojlovic et al., 2007**	USA	Cross-sectional study	General adults ICU.	ICU nurses (N = 866).	To evaluate the relationship between nurse–physician communication, healthy work environments, and the frequency of nursing-sensitive adverse events.	Self-reported frequency of ventilator-associated pneumonia, catheter-related sepsis, and medication errors.<br>Incidence of adverse events.	A strong association was found between healthy work environments and effective nurse–physician communication (p = 0.001). Improved communication correlated with fewer medication errors, identifying a direct process–outcome link related to patient safety and nursing-sensitive outcomes (NSOs).
**Alguire et al., 2014**	Canada	Cross-sectional study	Critical care units.	Critical care nurses (N = 207).	To evaluate critical care nurses' attitudes towards the role of families in nursing care.	Measures nurses' attitudes towards the role of families in care.<br>Families' Importance in Nursing Care- Nurses' Attitudes (FINC-NA) scale.	Nurses aged > 50 years and those with >5 years of ICU experience had significantly higher FINC-NA scores, reflecting more positive attitudes toward family involvement. Direct care nurses demonstrated lower FINC-NA scores compared to non-clinical staff (educators, managers).
**Lah et al., 2017**	USA	Retrospective cohort study	General ICU.	Adults patients (N = 1754).	To compare patient and family perceptions of ICU care.	Support of vital functions, monitoring of vital parameters and nursing care.<br>Hospital Consumer Assessment of Healthcare Providers and Systems (HCAHPS) scale; the Patient Perception of Quality (PPQ) scale.	Pearson correlation between HCAHPS and PPQ scores was moderate (*r* = 0.32; 95% CI: 0.28–0.37; *p* < 0.001). Stronger correlation was observed in patient-completed PPQ (*r* =0.45) compared to family-completed PPQ (*r* = 0.30). A discordance of 33% was noted between the two scales. The ICU length-of-stay/hospital length-of-stay ratio was significantly associated with differences in scores (*p* = 0.02).
**Cho et al., 2017**	South Korea	Quasi-experimental study	General ICU.	Adults patients (N = 84): Intervention group (N = 42), Control group (N = 42).	To evaluate the effects of aromatherapy with lavender essential oil on stress and sleep quality in ICU patients.	Aromatherapy with lavender essential oil combined with deep breathing exercises for 20 minutes nightly over 2 nights.<br>Perceived and objective stress indices, vital parameters, and sleep quality scales.	Patients in the intervention group reported significant reductions in perceived stress (F = 60.11, *p* < 0.001), objective stress (F = 25.65, *p* < 0.001), systolic blood pressure (F = 9.09, *p* < 0.001), diastolic blood pressure (F = 2.47, *p* = 0.046), and heart rate (F = 5.71, *p* < 0.001). Sleep quality significantly improved compared to the control group (F = 109.46, *p* < 0.001).

**Tayyib et al., 2016**	Australia	Systematic review and meta-analysis	Mixed ICUs	Various studies involving critically ill adult patients (N = 25).	To evaluate the effectiveness of nursing interventions for the prevention of pressure ulcers (Pus).	Pus prevention strategies, including silicone foam dressings, patient repositioning, specialized support surfaces, and nurse education.<br>Pus incidence; lesion staging.	Silicone foam dressings were associated with a significant reduction in pressure ulcer incidence (RR = 4.5–4.6; 95% CI: 0.05–0.31; $p < 0.00001$). Evidence for other interventions was limited or insufficient to demonstrate effectiveness.
**Akhond et al., 2017**	Iran	Parallel-group non-randomised clinical trial	General ICUs.	Adults patients (N = 120): Intervention group (N = 60); Control group (N = 60).	To evaluate the effectiveness of a structured pain monitoring protocol compared with usual care in reducing pain intensity.	Structured pain monitoring protocol.<br>Numeric Rating Scale (NRS).	Post-intervention, the intervention group reported significantly lower overall pain intensity compared with controls (3.2 ± 0.6 vs 7.2 ± 0.7, $p < 0.001$). Resting-state pain (2.6 ± 1.1 vs 5.1 ± 1.3, $p < 0.001$), suctioning pain (4.1 ± 1.1 vs 8.4 ± 0.9, $p < 0.001$), and dressing change pain (3.1 ± 1.0 vs 8.2 ± 0.9, $p < 0.001$) were all significantly reduced in the intervention group.
**D'Innocenzo et al., 2017**	Brasil	Cross-sectional study	General ICUs.	Adults patients (N = 304).	To identify the incidence and associated factors of adverse events (AEs).	Structured daily data collection.<br>Acute Physiologic Assessment and Chronic Health Evaluation (APACHE) II, Nursing Activities Score (NAS), Braden Scale, Glasgow Coma Scale.	The incidence of AEs was 12.8% (39/304), with pressure sores being the most frequent (43.6%), followed by catheter/tube losses. Patients with AEs were significantly older ($p < 0.001$), had longer ICU length of stay ($p < 0.001$), higher APACHE II ($p < 0.001$) and NAS scores ($p < 0.001$), and lower Braden ($p < 0.001$) and Glasgow scores ($p < 0.001$). No association was found with staffing levels.
**Richardson et al., 2017**	United Kingdom	Cross-sectional study	General ICUs.	Adults patients (N = 1015).	To evaluate the effectiveness of a pressure ulcer (PUs) prevention bundle in reducing PUs incidence.	Implementation of a PUs prevention bundle.<br>Braden Scale.	PUs incidence decreased significantly from 8.08/100 admissions to 2.97/100, representing a 63% relative reduction over four years.
**Köse et al., 2016**	Turkey	Cross-sectional study	General adults ICUs.	ICU nurses (N = 73).	To identify the knowledge of ICU nurses regarding the prevention of pressure ulcers (PUs).	PUs preventive interventions information Form (based on EPUAP–NPUAP Quick Reference Guide).<br>Braden Scale.	Significant associations were found with age ($\chi^2 = 6.066$, $p = 0.048$), education ($\chi^2 = 6.453$, $p = 0.040$), experience ($F = 4.189$, $p = 0.019$), ICU tenure ($F = 5.305$, $p = 0.007$), and education type ($F = 4.037$, $p = 0.016$). No significant differences emerged by gender, recent education, or unit type ($p > 0.05$).
**Mendes et al., 2016**	Brasil	Qualitative phenomenological study	General adults ICUs.	Family members of ICU patients (N = 21).	To interpret how family members of ICU patients experience the sensitivity of professionals in addressing their information needs.	Nursing advocacy.<br>Narrative analysis of interviews using phenomenological reflection.	Two experiential dimensions were identified: *presence of information*, associated with feelings of trust, comfort, and security; and *absence of information*, linked to anxiety, insecurity, and distress. Findings highlighted the crucial role of nursing communication in meeting families' informational needs and supporting their psychological well-being in ICU settings.

**Rosenthal et al., 2012**	Multi-country: Argentina, Brasil, China, Colombia, Costa Rica, Cuba, India, Lebanon, Macedonia, Mexico, Morocco, Panama, Perù, Philippine, Turkey.	Prospective cohort study	General adult ICUs across multiple countries.	Adults patients bladder catheter users (N = 56429).	To evaluate the effectiveness of a multidimensio -nal infection control strategy in reducing catheter-associated urinary tract infections (CAUTI).	Implementation of a CAUTI prevention bundle, including nursing education, process surveillance, feedback on preventive performance.<br>CAUTI rates per 1,000 catheter-days.	The CAUTI rate decreased significantly from 7.86 to 4.95 per 1,000 catheter-days across 253,122 urinary catheter-days, corresponding to a 37% reduction (RR=0.63, 95% CI: 0.55–0.72).
**Rogenski et al., 2012**	Brasil	Prospective cohort study	General ICU.	Adults patients (N = 78).	To evaluate the effectiveness of a nurse-led pressure ulcers (PUs) prevention protocol in reducing the incidence of PUs in ICU patients.	Implementation of a PUs prevention protocol including systematic risk assessment and preventive nursing measures; PUs incidence.	The incidence of PUs decreased from 41.02% before the protocol (2009) to 23.1% post-protocol implementation, demonstrating a significant reduction.
**Werli-Alvarenga et al., 2011**	Brasil	Prospective cohort study	General ICU.	Adults patients (N = 254).	To evaluate the risk exposure to ocular surface disease (OSD) and ocular outcomes in ICU patients in relation to nursing eye care practices.	Daily ocular assessment and corneal clinical observation.<br>Incidence OSD.	Corneal injuries occurred in 59.4% of patients, punctate injuries in 55.1%, and corneal ulcers in 11.8%, with a mean onset time of 8.9 days. Risk factors for punctate injuries included prolonged ICU stay, ventilatory support, edema, and blink rate < 5/min, while risk factors for corneal ulcers included lower Glasgow Coma Scale scores and ocular globe exposure.
**Williams et al., 2010**	Australia	Retrospective cohort study	General ICUs.	Adults patients (N = 22298).	To evaluate the association between ICU length of stay (LOS) and both in-hospital and long-term mortality.	Usual care.<br>ICU LOS; in-hospital; long-term mortality.	In-hospital mortality was not independently associated with ICU LOS after adjustment. Long-term mortality showed a slight increase in risk, with ICU LOS explaining 2.3% of χ^2 variability compared to age (35.8%) and comorbidities (18.6%).
**Da Silveira et al., 2018**	Brasil	Retrospective cohort study	General ICUs.	Adults patients (N = 249)	To evaluate functional status before ICU admission and immediately after discharge.	Usual care.<br>Barthel Index (BI) and Katz Index (KI).	Both scales detected significant declines in ADLs, with a median BI drop of 44% and a median KI drop of 55%. However, the BI showed greater item variation and discrimination in IRT analysis, indicating its superior suitability for functional assessment of ICU survivors.

**de Medeiros Araújo et al., 2018**	Brasil	Cross-sectional study	General ICU.	Adults patients (N = 30).	To evaluate the nursing diagnosis of dry eye risk and ocular dryness in ICU patients.	Daily ocular assessment and corneal clinical observation.<br><br>NANDA-I Taxonomy II criteria; Schirmer test (<10 mm); clinical signs of ocular dryness.	The prevalence of dry eye risk was 21.7% (13/60 eyes), while ocular dryness was observed in 78.3% (47/60 eyes). Significant risk factors for dry eye included mechanical ventilation ($p = 0.021$), neurological lesions with sensory motor loss ($p = 0.021$), Schirmer test results (median 18 mm, $p < 0.001$), and hospital stay duration (median 2 days, $p = 0.020$). Predictors of ocular dryness were Schirmer test median 4 mm ($p < 0.001$), hospital stay (median 6 days, p = 0.020), and mechanical ventilation (44.7%, $p = 0.041$).
**de Araújo et al., 2018**	Brasil	Cross-sectional study	General adult ICU.	Adults patients (N = 98).	To evaluate the risk for ocular damage, specifically dry eye, in ICU patients using clinical criteria from NANDA-I Taxonomy.	Daily ocular assessment and corneal clinical observation.<br><br>NANDA-I Taxonomy II: “Risk for dry eye”; Schirmer I test.	The most frequent risk factors identified were environmental/care-related (100%), aging (55.1%), mechanical ventilation (50%), and female sex (49%). Commonly associated drugs included gastric protectors (74.5%), antibiotics (64.3%), anticoagulants (50%), and opioids (36.7%).
**Elliott et al., 2016**	Australia	Prospective cohort study	General ICU.	Adults patients (N = 222).	To observe the factors influencing the development of post-traumatic stress disorder (PTSD) and related psychological outcomes after ICU admission.	Usual care.<br><br>Posttraumatic Stress Disorder Checklist–Specific (PCL-S); Depression, Anxiety, Stress Scales-21 (DASS-21); self-reported sleep quality.	At 6-month follow-up, 30% of patients reported depression and 13.5% exhibited PTSD symptoms. The regression model ($R^2 = 0.682$) identified independent predictors, including ICU experiences, baseline pain, stress/depression, and ongoing sleep quality.
**Ohta et al., 2014**	Japan	Prospective cohort study	General ICU.	Adults patients (N = 459).	To observe and report the incidence of adverse drug events (ADEs) and their association with patient outcomes in ICU settings.	Systematic monitoring and documentation ADEs.<br><br>ADEs incidence.	ADE incidence was 99 events in 70 patients (15%), corresponding to 30.6 per 1,000 patient-days or 21.6 per 100 admissions. ICU mortality was 16%, with higher mortality among ADE-exposed patients (17%) compared to non-ADE patients (7%, $p = 0.003$), excluding early deaths (< 3 days). ICU length of stay was significantly longer among patients with ≥1 ADE (13 days) compared to non-ADE patients (2 days, $p < 0.0001$). Adjusted analyses indicated ADEs were significantly associated with prolonged ICU stay but not independently predictive of mortality after controlling for illness severity.
**Seynaeve et al., 2011**	Belgium	Cross-sectional study	General ICU.	ICU patients (N = 79).	To evaluate the characteristics of adverse drug events (ADEs) and determine the influence of disease severity and nursing	Monitoring of ADEs.<br><br>Global Trigger Tool adapted for ICU (severity algorithm); illness severity and nursing workload assessed using validated scoring systems.	ADEs were identified on 175 of 1,009 ICU-days, with a total of 230 ADEs. The majority (96%) resulted in temporary harm, while 4% led to complications. Days with ADEs were associated with significantly higher mean illness severity and greater nursing workload, underscoring the role of patient complexity and staff demands in ADE occurrence.

					workload on the prevalence of these events.		
**Jeong et al., 2010**	South Korea	Non-randomised clinical trial	General ICU.	Female patients bladder catheter users (N = 97).	To compare the effectiveness of four perineal care agents in preventing catheter-associated urinary tract infections (CAUTIs).	Daily perineal care using soap and water, skin cleansing foam (Menalind), 10% povidone-iodine, or normal saline.<br>CAUTI incidence per 100 catheter-days.	Cumulative CAUTI rates were 3.18 (week 1), 3.31 (week 2), and 3.04 (week 4) per 100 catheter-days. No significant differences were observed in CAUTI hazard ratios among the four agents across all time points.
**Özcaka et al., 2012**	Turkey	Randomised controlled trial	General ICU.	Mechanically ventilated patients (N = 61): Intervention group (N = 29), Control group (N = 32).	To evaluate the effectiveness of oral care with chlorhexidine vs saline in preventing ventilator-associated pneumonia (VAP).	Oral care protocol with four daily swabbings using 0.2% chlorhexidine gluconate vs saline solution.<br>Incidence of VAP.	VAP incidence was significantly lower in the chlorhexidine group (41.4%) compared to the saline group (68.8%) (OR = 3.12, 95% CI: 1.09–8.91, p = 0.03). Mean time to VAP onset was 6.8 days, with no significant differences in periodontal indices, pathogens, or mortality between groups.
**Forster et al., 2008**	Canada	Prospective cohort study	General ICU.	Adults patients (N = 207).	To evaluate the occurrence of adverse events (AEs) in the ICU, their preventability, and their association with patient outcomes.	Daily monitoring and multidisciplinary review of all clinical events to identify ICU-based AEs.<br>In-hospital mortality and length of stay.	In-hospital mortality was 25% (95% CI: 19–31). Neither AEs nor preventable AEs were associated with time to death (HR = 0.93 vs 0.72, not significant). Median ICU length of stay was 15 days (IQR: 8–34). ICU AEs significantly delayed discharge (HR = 0.50, 95% CI: 0.31–0.81), with preventable AEs showing a similar effect (HR = 0.46, 95% CI: 0.23–0.91).
**Macht et al., 2011**	USA	Retrospective cohort study	General ICU.	Patients extubated after invasive mechanical ventilation (N = 446).	To evaluate the prevalence of post-extubation dysphagia and its association with pneumonia and clinical outcomes.	To administer the Bedside Swallowing Evaluation Test (BSE) performed after extubation.<br>Incidence of post-extubation dysphagia and its severity staging.	Dysphagia was identified in 84% of patients (n = 374). Mechanical ventilation lasting longer than 7 days was independently associated with moderate/severe dysphagia (44%, aOR = 2.84, p < 0.01). Moderate/severe dysphagia was in turn associated with poorer outcomes, including pneumonia, reintubation, and in-hospital mortality (aOR = 3.31, p < 0.01).
**Gonzales et al., 2013**	Canada	Cross-sectional study	Adult ICUs (not further specified)	Patients with central venous catheters (N = 47)	To evaluate self-reported prevention practices and monitor the incidence of central line-associated bloodstream infections (CLABSI) in ICU patients.	Implementation and monitoring of catheter insertion bundles.<br>CLABSI rates.	ICUs that monitored compliance with catheter insertion bundles demonstrated a significantly greater reduction in CLABSI rates compared to units without monitoring, even after adjusting for teaching status and bed count (p = 0.036).
**de Groot et al., 2011**	Netherlands	Prospective case–control study	General ICUs.	Mechanically ventilated patients (N = 370): case group (N = 74); control group	To observe whether unplanned extubation (UE) is associated with increased mortality and morbidity among mechanically ventilated ICU patients.	Monitoring of UE events.<br>In-hospital mortality; length of stay (LOS).	UE patients had lower hospital mortality compared to controls (19% vs 32%, p = 0.028; adjusted OR = 0.5, 95% CI: 0.28–1.00). Among UE cases, 47% required reintubation. Non-reintubated patients had significantly shorter ICU and hospital LOS and lower mortality.

				(N = 296).			
**Tabah et al., 2010**	France	Prospective cohort study	General ICUs.	Patients aged 80 years or older<br>(N = 106).	To describe self-sufficiency and quality of life (QoL) one year after ICU discharge in elderly patients.	Assessment of functional independence at ICU admission and one year after discharge.<br><br>Katz Index of Activities of Daily Living (ADLs).	Among 23 survivors, no changes in Katz ADL scores were observed from baseline (median = 6 at both time points). QoL scores for physical, sensory, self-sufficiency, and social participation domains were slightly lower than in the general population, while psychological health, social relationships, environment, and attitudes toward death were equal to or better than those of matched controls.
**Kim et al., 2023**	South Korea	Non-randomised controlled trial	Cardiothoracic and General ICUs.	ICU patients on ECMO<br>(N = 52):<br>Intervention group<br>(N = 26);<br>Control group<br>(N = 26).<br><br>ICU nurses<br>(N = 56):<br>Intervention group<br>(N = 28);<br>Control group<br>(N = 28).	To evaluate the effectiveness of an evidence-based ECMO nursing protocol on patient complications and nurse-related outcomes.	Implementation of an evidence-based ECMO nursing protocol, including circuit management.<br><br>Infection rate; pressure injury rate; nurse satisfaction; empowerment;<br>performance (self-reported).	No differences were observed in physiological indicators (vital signs, pump parameters). However, infection rates ($p = 0.026$) and pressure injury rates ($p = 0.041$) were significantly lower in the intervention group. Nurse-related outcomes, including satisfaction, empowerment, and performance, were significantly higher in the intervention group compared to controls (all $p < 0.001$).
**Padilla et al., 2023**	Chile	Retrospective cohort study	General ICUs.	Adults critically patients<br>(N = 311).	To determine the association between nursing workload, patient severity of illness, and mortality in ICUs.	Estimation of the nursing workload during the shift and assessment of the severity of the patient's condition within the first 24 hours after admission to the intensive care unit.<br><br>Therapeutic Intervention Scoring System-28 (TISS-28); The Acute Physiology; Chronic Health Evaluation II (APACHE II)	TISS-28 correlated with APACHE II ($r = 0.359$, $p < 0.001$) and ICU length of stay ($r = 0.146$, $p < 0.05$). ICU mortality was 22.8%, and hospital mortality was 14.2%. Multivariable analysis showed ICU mortality increased by 10.3% per APACHE II point (OR = 1.109; 95% CI: 1.065–1.155, $p < 0.001$), but decreased by 4.5% per TISS-28 point (OR = 0.955; 95% CI: 0.914–0.999, $p = 0.045$). Hospital mortality increased by 7.6% per ICU length of stay day (OR = 1.079; 95% CI: 1.034–1.126, $p < 0.001$). APACHE II and TISS-28 were not significantly associated with hospital mortality.
**Jin et al., 2018**	South Korea	Retrospective cohort study	General ICUs.	Adults patients<br>(N = 148):<br>before Nutrition Support Team group<br>(N = 75);<br>after Nutrition Support Team group<br>(N = 73).	To evaluate the effects of implementing a multidisciplinary Nutrition Support Team (NST) on nutritional adequacy, clinical outcomes, and mortality in ICU patients.	Implementation of NST with monitoring of nutritional adequacy.<br><br>Percentage of goal kcal and protein intake, serum albumin levels, lymphocyte count.	After NST implementation, goal kcal increased from 66.9 ± 25.9% to 86.2 ± 27.5% ($p < 0.001$), and goal protein from 67.0 ± 29.9% to 81.7 ± 30.7% ($p < 0.05$). Increased calorie achievement was associated with shorter ICU and hospital length of stay (both $p < 0.05$). Each 1% increase in kcal intake reduced mortality risk (OR = 0.977; 95% CI: 0.959–0.996; $p = 0.016$).
**Damico et al., 2018**	Italy	Quasi-experimental study	General ICUs.	Adults patients<br>(N = 587):	To evaluate the impact of a nurse-driven pain	Implementation of nusing protocol guiding priority of	Implementation of the protocol significantly reduced the proportion of patients recalling severe pain at one

				Pre-intervention group (N = 370); Post-intervention group (N = 217).	management protocol on sedation and analgesia use, as well as long-term pain recall among ICU patients.	analgesia over sedation for moderate to severe pain.<br>Behavioral Pain Scale (BPS); Numeric Rating Scale (NRS).	year post-discharge (*p* = 0.037). Sedative use decreased (p < 0.001), while the administration of “as needed” anti-inflammatories and analgesics increased (*p* = 0.0028). No significant change was observed in strong opioid usage.
**Haghighi et al., 2016**	Iran	Randomised controlled trial	General ICUs.	Mechanically ventilated patients (N = 100): intervention group (N = 60); control group (N = 40).	To evaluate the effectiveness of a systematic, nurse-delivered oral care program on oral health and prevention of ventilator-associated pneumonia (VAP).	Implementation of a structured oral care program administered by nurses.<br>Beck Oral Assessment Scale (BOAS); mucosal–plaque index; Clinical Pulmonary Infection Score (CPIS).	Oral health improved significantly in the intervention group, with better BOAS scores (days 1–5, *p* < 0.001) and reduced mucosal–plaque index (days 3–5, *p* < 0.001). Although VAP incidence was lower in the intervention group (Day 5: 10% vs 14%), the difference was not statistically significant (*p* = 0.059).
**Chipps et al., 2016**	USA	Randomised controlled trial	General ICUs.	ICU patients (N = 74); Intervention group (N = 37); Control group (N = 37).	To evaluate the effectiveness of a structured, nurse delivered oral care protocol on oral health, microbial colonization, and patient satisfaction in ICU patients.	Implementation of nursing oral care protocol including tooth brushing, tongue scraping, flossing, mouth rinse, and lip care.<br>Revised Oral Health Assessment Tool (R-THROAT); MSSA/MRSA oral cultures.	The intervention group showed significantly greater oral health improvement (+1.97 vs +0.87; *p* = 0.04), with marked gains in tongue and mouth comfort domains. No differences were found in MSSA/MRSA colonization between groups. Patient satisfaction was higher in the intervention group.
**Dang et al., 2013**	USA	Narrative review	General ICUs.	Unspecified number of studies reviewed.	To evaluate the impact of the ABCDE bundle, particularly the “E” (early mobility) component—on functional outcomes, duration of mechanical ventilation, and length of stay.	Nurse participation in the ABCDE bundle with emphasis on early mobility interventions (physiotherapy collaboration, head-of-bed positioning, early mobilization during mechanical ventilation).<br>Duration of mechanical ventilation; ICU and hospital length of stay (LOS); functional status.	Evidence showed improved independent functional status at discharge (OR = 2.5; 95% CI: 1.2–5.1; *p* < 0.05). Other included studies reported reductions in mechanical ventilation duration, ICU and hospital LOS, and improved muscle strength and physical function with early mobility interventions.
**Chavez et al., 2015**	USA	Case series	Cardiothoracic ICU.	Adults patients with ECMO/VAD support (N = 3).	To evaluate the feasibility and safety of a Progressive Mobility (PM) guideline for critically ill patients supported with ECMO/VAD.	Implementation of a nurse-led Progressive Mobility (PM) guideline, structured as sequential milestones from passive movements to ambulation.<br>ECMO/VAD device-related adverse events; functional level.	All patients were successfully mobilized to ambulation during ECMO/VAD support. No serious or device-related adverse events occurred during initial mobility sessions. The intervention demonstrated feasibility, with patients achieving higher functional levels (including ambulation) during ICU stay.

**Black et al., 2011**	Northern Ireland	Quasi-experimental study	General ICU.	Adults patients (N = 170): Intervention group (N = 87); Control group (N = 83).	To evaluate the impact of nurse-facilitated family participation in psychological care on delirium incidence and psychological recovery in critically ill patients.	Nurse-facilitated family participation in psychological care during ICU stay versus standard care.<br><br>Therapeutic Intervention Scoring System (TISS-28), Intensive Care Delirium Screening Checklist (ICDSC), Sickness Impact Profile (SIP).	No significant difference in delirium incidence between groups (7-day comparison, $p = 0.34$). Patients in the intervention group showed significantly better psychological recovery, with improved SIP scores at 4, 8, and 12 weeks post-admission ($p = 0.001$).
**Bliss et al., 2011**	USA	Prospective cohort study	Surgical ICU	Adults critically ill patients (N = 45).	To evaluate the incidence, severity, and risk factors of incontinenceassociated dermatitis (IAD).	Serial daily perineal skin assessments.<br><br>IAD incidence and severity scoring.	IAD incidence was 36%, with a median time to onset of 4 days (range 1–6). At ICU discharge, 81% of cases persisted (median duration 7 days, range 1–19). Severity distribution: mild erythema 13%, moderate 11%, severe 4%; skin denudement present 9% of observation periods. Frequent loose/liquid stools and diminished cognitive awareness were significantly associated with earlier IAD development.
**Choi et al., 2008**	USA	Narrative literature review	General ICUs.	ICU patients on prolonged mechanical ventilation ≥ 21 days (N = 10 studies)	To evaluate the effectiveness of mobility interventions in improving outcomes for ICU patients requiring prolonged mechanical ventilation.	Mobility interventions included whole-body physical therapy, electrical stimulation, arm exercise, and inspiratory muscle training.<br><br>Muscle strength; functional status; duration of prolonged mechanical ventilation; ICU and hospital length of stay (LOS).	Mobility interventions were associated with improved muscle strength and functional outcomes, as well as reductions in ICU and hospital LOS ($p < 0.05$). These findings highlight the relevance of structured mobility protocols as nurse-sensitive interventions in PMV care.
**Grogan et al., 2002**	USA	Cross-sectional study	General ICUs.	Patients with fecal incontinence (N = 22).	To evaluate the effectiveness of a nasopharyngeal airway ("rectal trumpet") used as a fecal containment device in ICU patients with fecal incontinence.	Insertion and monitoring of a rectal trumpet for fecal containment.<br><br>Stool containment grade, skin integrity; patient comfort.	Stool containment was achieved or improved in 100% of cases. Among patients with pre-existing skin injury, 90% showed healing or restoration of integrity. Discomfort was reported during insertion in 41% of patients, but 86% reported no discomfort while the device was in place. Findings emphasize the role of ICU nurses in implementing fecal containment strategies to prevent skin breakdown and enhance patient comfort.
**Wlodarczyk-Abou et al., 2023**	Finland	Prospective cohort study	General ICUs.	Critically ill adult patients (N = 711).	To evaluate the incidence and risk factors of pain during a 10-day ICU stay.	Systematic pain evaluation during ICU stay.<br><br>Numeric Rating Scale (NRS) and Verbal Rating Scale (VRS) for communicating patients; Critical Care Pain Observation Tool (CPOT) for non-communicating patients.	76% of patients experienced moderate-to-severe pain during ICU stay. High-risk groups included patients <64 years, females, and those with a history of chronic pain. No significant differences in pain incidence were found between surgical and non-surgical patients ($p > 0.05$). Findings highlight the central role of nurses in routine and structured pain assessment to identify at-risk groups and optimize pain management strategies.

**Watanabe et al., 2022**	Japan	Prospective cohort study	General ICUs.	Adults discharged patients<br>(N = 192):<br>Early mobilization cohort<br>(N = 107);<br>Non- early mobilization cohort<br>(N = 85).	To evaluate the effect of early mobilization (EM) within 72 hours of ICU admission on the incidence of psychiatric symptoms after ICU discharge.	Implementation of an early mobilization protocol (sitting at edge of bed or higher within 72 h of ICU admission), facilitated by nurses in collaboration with the care team.<br>Standardized psychiatric symptom assessment tools for depression; anxiety.	Psychiatric symptom incidence was significantly lower in the EM cohort compared to the non-EM cohort (25% vs 51%, $p = 0.008$). Adjusted analyses confirmed EM as a protective factor against psychiatric symptoms (aOR = 0.27, $p = 0.032$; RR = 0.49, 95% CI: 0.29–0.83). Sensitivity analyses, including Inverse Probability of Treatment Weighting, yielded consistent results (aOR ≈ 0.28–0.49, p = 0.008–0.046). Findings highlight the impact of nurse-driven mobilization strategies in improving post-ICU psychological outcomes.
**Fuest et al., 2021**	Germany	Prospective cohort study	General ICUs.	Frail and non-frail adult patients<br>(N = 1172).	To evaluate functional trajectories of frail versus non-frail ICU patients during hospitalisation.	Nursing-sensitive monitoring included systematic assessment of frailty and functional outcomes.<br>Clinical Frailty Scale (CFS) and "Mobility" and "Transfer" domains of the Barthel Index (MTB).	Frail patients had lower baseline functional status compared to non-frail peers. After adjustment, the odds of MTB deterioration by hospital discharge were not significantly different (OR = 1.3; 95% CI: 0.8–1.9; $p = 0.301$). Interestingly, frail patients exhibited less early MTB decline by ICU discharge (OR = 0.2, $p < 0.001$). Sensitivity analyses confirmed robustness across all patients and survivors only. Results highlight the role of nursing-led functional monitoring, showing that frailty does not preclude recovery potential when rehabilitation trajectories are actively supported.
**Baby et al., 2021**	India	Prospective observational study	General ICUs.	Adults patients<br>(N = 250).	To evaluate the incidence, clinical course, and impact of ICU-acquired weakness (ICUAW) on ICU length of stay and outcomes.	Nursing sensitive monitoring of neuromuscular status.<br>Medical Research Council (MRC) score to detect and follow ICUAW.	Incidence of ICUAW was 50% in non-survivors versus 7.83% in survivors. Patients with ICUAW had longer ICU stays (10.93 vs 5.03 days), more ventilation days (9.27 vs 3.87), and significantly higher ICU mortality (50% vs 7.83%). Findings emphasize the importance of systematic nursing surveillance for early detection of ICUAW, enabling timely rehabilitation and preventive strategies.
**Zhou et al., 2021**	USA	Retrospective cohort study	Medical, surgical, and medical-surgical ICUs	Mechanically ventilated adults patients<br>(N = 676).	To measure the incidence and types of patient–ventilator asynchrony (PVA), identify risk factors for its development, and explore its relationship with patient outcomes.	Nursing surveillance of pressure/volume waveforms was used to detect PVA events, focusing on double triggering and flow starvation. Nursing-sensitive outcomes included monitoring hospital-free days	Incidence of PVA was 24% overall, with double triggering (13%) and flow starvation (10%) as the most frequent types. PVA was associated with fewer hospital-free days (15.2 vs 19.7) but not with adjusted hospital mortality (p > 0.05). Results highlight the critical nursing role in ventilator waveform monitoring to identify asynchronies and mitigate their clinical impact.

						and mortality in relation to PVA detection.	
**Boniatti et al., 2021**	Brasil	Prospective cohort study	General adults ICUs.	Family members of ICU patients (N = 131).	To evaluate the association between resilience and caregiver burden, anxiety, and depression among family members of ICU patients.	Psychosocial support interventions provided to families to mitigate caregiver burden and psychological distress.<br>Connor-Davidson Resilience Scale; the Duke University Religion Index (DUREL); the Hospital Anxiety and Depression Scale (HADS); the Zarit Burden Interview.	Clinically significant caregiver burden was identified in 72.4% (Zarit ≥ 21). Resilient caregivers reported significantly lower anxiety (mean 9.0 vs 11.3; p = 0.011), depression (6.4 vs 9.1; *p* < 0.001), and burden (27.5 vs 35.7; *p* = 0.015). Resilience was independently associated with reduced caregiver burden, anxiety, and depression, underscoring the need for nursing interventions that foster resilience in family caregivers of ICU patients.
**Li et al., 2021**	China	Prospective cohort study	General ICUs.	Adults patients (N = 940):<br>Physical prophylaxis (N = 635);<br>Drug prophylaxis (N = 305).	To evaluate the incidence of deep venous thrombosis (DVT) in critically ill patients receiving routine thromboprophylaxis and to identify associated risk/protective factors.	The consistent application of thromboprophylaxis measures as guided by local standards of care.<br>DVT incidence; laboratory monitoring (D-dimer levels).	DVT incidence was 6.2% (58 patients). Thirty-six patients with DVT received anticoagulant therapy (all Low Molecular Weight Heparin). Independent associations with DVT included elevated D-dimer levels (OR = 1.256, 95% CI: 1.132–1.990), while both basic prophylaxis (OR = 0.092, 95% CI: 0.016–0.536) and physical prophylaxis (OR = 0.159, 95% CI: 0.038–0.674) were protective. No significant differences were observed in short-term survival between DVT and non-DVT patients.
**Shinn et al., 2019**	USA	Prospective cohort study	General ICUs	Intubated (ETT) adults patients (N = 814): Adequate ETT size for height (N = 408);<br>Large ETT for height (N = 224);<br>Small ETT for height (N = 182).	To evaluate the incidence of upper airway injuries following prolonged endotracheal intubation and to identify associated risk factors.	Airway surveillance and monitoring of post-extubation complications through early assessment and reporting of upper airway lesions.<br>Flexible nasolaryngoscopy (≤ 36 hours after extubation); Clinical COPD Questionnaire (CCQ); Voice Handicap Index (VHI-10).	Acute laryngeal injury (ALgI) incidence was 57%, including mucosal ulceration (48%), granulation (19%), and subglottic ulceration/granulation (8%). Patients with ALgI had worse CCQ (median 1.05 vs 0.20; *p* < 0.001) and VHI-10 scores (median 2 vs 0; *p* = 0.005). Multivariable analysis identified larger ETT size, diabetes (OR = 3.14; *p* = 0.002), and higher BMI (OR = 3.09 per unit increase; *p* = 0.007) as independent risk factors.
**Haugdahl et al., 2018**	Norway	Qualitative hermeneutic phenomenological study	General ICU.	Family members of long-term adults patients (N = 13).	To explore family members' lived experiences of being present with long-term ICU patients and their perceived role in patient survival and recovery.	Facilitators of communication, emotional support and recognition of the central role of family presence and cooperation with relatives in daily care.<br>In-depth interviews analysed using a thematic alysis with a salutogenic and phenomenological framework.	Three themes emerged: (1) *A body at a breaking point* – patients oscillating between life and death; (2) *Family members' presence* – expressing love, interpreting cues, sustaining identity; (3) *Breaking through* – family engagement as a trigger for patient motivation, hope, and resilience, in collaboration with staff. Family presence was viewed as a health-promoting force that strengthened survival and recovery.

**Nedergaard et al., 2018**	Denmark	Mixed methods study	General ICU.	ICU survivors (N = 32); ICU nurses (N = 54); Anesthesiologists (N = 17).	To evaluate which outcomes are valued most by ICU survivors after discharge and to assess the alignment of provider awareness (nurses and physicians).	Improvement of cognitive and emotional recovery, reflecting the holistic approach to intensive care nursing.<br>Three-phase study: (1) qualitative interviews with survivors; (2) item reduction of 36 to 20 outcomes; (3) prioritization ranking by survivors, nurses, and physicians via individual rating tool.	ICU survivors prioritized functional physical recovery as the most important outcome. Nurses placed greater emphasis on cognitive and emotional domains, while anesthesiologists focused mainly on physical outcomes, aligning more closely with survivors. Findings highlight the distinct nursing role in addressing psychological and cognitive recovery as core dimensions of post-ICU care.
**López et al., 2018**	Spain	Prospective cohort study	General ICUs.	Severely traumatised, mechanically ventilated ICU patients. (N = 124).	To evaluate pain assessment in critically ill patients during nursing procedures (suctioning and mobilization) across different ICU time points.	To evaluation pain during tracheal suctioning and mobilization at days 1, 3, and 6 of ICU stay.<br>European Society of Clinical Microbiology and Infectious Diseases (ESCID) scores Behavioral Pain Scale (BPS)**.**	ESCID scores significantly increased during suctioning and mobilization (*p* < 0.01), confirming these procedures as painful events. High inter-rater reliability (Kappa > 0.84, "almost perfect") demonstrated the tool's consistency and applicability in nursing practice. Results highlight the central role of nurses in early detection and management of pain during routine ICU care.
**Zhao et al., 2017**	China	Randomised controlled trial	General ICUs.	Adults survivors (N = 71): Intervention group (N = 35); Control group (N = 36).	To evaluate the effect of an early structured cognitive intervention on cognitive recovery after ICU discharge.	Delivery and monitoring of the early cognitive intervention training programme, which included keyboard play, clock drawing and simple language learning for 2 months. Montreal Cognitive Assessment (MoCA) at baseline and at 2 months.	Cognitive impairment incidence at 2 months was significantly lower in the intervention group (38.8%) compared to controls (60.6%, $\chi^2 = 6.321$, $p = 0.015$). Mean MoCA scores were higher in the intervention group (26.73 ± 1.92) than in the control (24.95 ± 2.26; $p < 0.05$). Subdomains (executive, memory, attention, orientation) showed significant improvements in the intervention group ($p < 0.05$), underscoring the importance of nurse-facilitated cognitive rehabilitation in ICU survivors.
**Bosca et al., 2013**	Romania	Prospective cohort study	General ICUs.	Mechanically ventilated adult patients ≥ 48 hours (N = 30).	To evaluate the effectiveness of 0.5% chlorhexidine oral decontamination administered at different time intervals in reducing oral colonisation and mucosal plaque.	Oral decontamination with 0.5% chlorhexidine every 6 h or 12 h.<br>Mucosal Plaque Score (MPS); oral culture swabs to assess colonisation.	Mean MPS was comparable between the 6 h group (3.8–6) and 12 h group (3.6–5; $p = 0.898$). Colonization was less frequent in the 12 h group (40% vs 60%), though differences were not statistically significant ($p = 0.523$). Importantly, MRSA colonization was significantly lower in the 6 h group ($p = 0.032$), suggesting potential benefits of more frequent oral care by nursing staff.

**Laupland et al., 2012**	France	Retrospective cohort study	General ICUs.	Adults patients excluding patients in therapeutic (N = 10962).	To evaluate the determinants of temperature abnormalities on ICU admission (< 24 h) and their impact on outcomes in critically ill patients.	Systematically monitored body temperature within the first 24 h and classifying patients into hypothermia (mild/moderate/severe), fever (mild/high), or normothermia.<br>Measurement of body temperature in °C.	Hypothermia prevalence: mild (10%), moderate (5%), severe (<1%). Fever prevalence: mild (21%), high (5%). Normothermia in 55%. Hypothermia independently predicted increased mortality, particularly in medical ICU patients, while fever/shivering showed no significant association with mortality after adjustment.
**Black et al., 2011**	Northern Ireland	Quasi-experimental study	General ICUs.	Adults patients (N = 170): Intervention group (N = 87), Control group (N = 83).	To evaluate the impact of nurse-facilitated family participation in psychological care during ICU stay.	Nurse-led facilitation of family psychological care.<br>Therapeutic Intervention Scoring System-28 (TISS-28); Intensive Care Delirium Screening Checklist (ICDSC), and Sickness Impact Profile (SIP).	No significant reduction in delirium incidence (ICDSC) during ICU stay. The intervention group demonstrated significantly better psychological recovery (SIP scores) at 4, 8, and 12 weeks post-admission compared to controls.
**Banerjee et al., 2011**	USA	Narrative review	Adul ICU.	Not specified (narrative synthesis of studies)	To evaluate evidence on sedation protocols, delirium monitoring, and early mobilization in ICU trauma populations.	Protocolized sedation interruption; minimisation of benzodiazepine use; systematic monitoring of delirium and implementation of early mobilisation protocols.<br>Intensive Care Delirium Screening Checklist (ICDSC); The Confusion Assessment Method for ICU (CAM – ICU).	Findings highlight that structured sedation interruption, consistent delirium screening, reduced benzodiazepine use, and early mobilization significantly improve patient outcomes by reducing delirium duration, mechanical ventilation days, and ICU length of stay.
**Girard et al., 2010**	USA	Prospective cohort study	Medical ICU.	Mechanically ventilated patients, (N = 126).	To evaluate the impact of delirium duration on long-term cognitive outcomes.	Daily delirium assessment, sedation level monitoring.<br>The Confusion Assessment Method for ICU (CAM–ICU), Richmond Agitation-Sedation Scale (RASS) and neuropsych tests.	Duration of delirium was an independent predictor of poorer cognitive performance at 3 months (p = 0.02) and 12 months (p = 0.03) post-discharge. Mechanical ventilation duration was not associated with long-term cognitive impairment.
**Laupland et al., 2008**	Canada	Retrospective cohort study	Mixed medical–surgical ICUs.	Adult patients (N = 20466).	To describe the onset of fever in critically ill patients and assess its effect on ICU outcomes.	Monitoring of temperature and management of symptoms associated with fever.<br>Incidence of fever (> 38.3°C).	Cumulative incidence of fever was 44%, with high fever in 8%. Incidence density was 24.3 fever days per 100 ICU-days, high fever 2.7/100 ICU-days. High fever was linked to significantly higher ICU mortality, while routine fever was common and varied by patient subgroup. Mortality rates: febrile vs afebrile 13% vs 12% (p = 0.08, not significant); high fever vs no high fever 20.3% vs 12% (p < 0.0001).

**Metheny et al., 2006**	USA	Prospective cohort study	Medical and surgical ICUs, academic teaching hospital.	Mechanically ventilated, tube-fed adult patients.<br>(N = 360).	To evaluate the frequency of aspiration events and their relationship with pneumonia in critically ill, tube-fed patients.	Endotracheal aspiration.<br><br>Aspiration measured via pepsin-positive tracheal secretions; Clinical Pulmonary Infection Score (CPIS).	1.3% of tracheal secretions tested positive for pepsin; 88.9% of patients had ≥1 aspiration event. Pneumonia incidence rose from 24% on day 1 to 48% by day 4. Aspiration was a significant risk factor for pneumonia ($p < 0.001$). Both aspiration and pneumonia were associated with longer ICU length of stay and prolonged mechanical ventilation ($p < 0.01$).
**Blot et al., 2005**	Belgium	Retrospective cohort study	Medical, surgical, ICUs, university hospital.	Adults patients with central venous catheters<br>(N = 491): Catheter-related bloodstream infections<br>(N = 176);<br>Without infection<br>(N = 315).	To evaluate the clinical outcomes associated with nosocomial central venous catheter–related bloodstream infections (CR-BSI).	Management of central venous catheters.<br><br>Mortality; in-hospital length of stay (LOS); duration of mechanical ventilation.	In-hospital mortality was comparable between groups (CR-BSI 27.8% vs. controls 26.0%), with an attributable mortality of only 1.8% (95% CI: –6.4% to 10.0%). However, CR-BSI was associated with significantly longer mechanical ventilation, prolonged ICU and hospital stays, and increased use of ICU resources.
**Hughes et al., 2001**	United Kingdom	Retrospective cohort study	General ICUs, university hospital.	Patients with length of stay (LOS) ≥ 30 days<br>(N = 323).	To evaluate outcomes for ICU patients with long stays.	Usual care.<br><br>Hospital survival, ICU occupancy (bed-days).	Patients with prolonged ICU stays represented only 1.6% of total admissions but accounted for 15.7% of ICU bed-days. Hospital survival among this group was 59.9%, highlighting the disproportionate resource use associated with long-stay ICU patients.
**Rello et al., 2000**	Spain	Prospective cohort study	General adult, tertiary hospital.	Adults patients with central venous catheters<br>(N = 98):<br>Catheter-related infections cohort<br>(N = 49);<br>Non-infection cohort<br>(N = 49).	To evaluate the impact of central venous catheter-related infections (CRIs) on patient outcomes.	Management of central venous catheters.<br><br>Diagnosis of CRIs (CDC criteria); hospital mortality; length of stay (LOS).	Hospital mortality was not significantly different between groups (CRI: 22.4% vs non-CRI: 34.7%, $p > 0.20$). However, among survivors, CRI was associated with significantly longer hospital stay (+19.6 days; 95% CI: 1.1–40.4), underlining the burden of CRIs on resource use.
**Kollef et al., 1997**	USA	Randomised controlled trial	Medical, surgical ICUs.	Mechanically ventilated adults patients<br>(N = 357):<br>Intervention group<br>(N = 179);<br>Control group<br>(N = 178).	To evaluate the effectiveness of protocol-directed weaning by nurses and respiratory therapists compared with physician-directed weaning.	Ventilatory weaning led by nurses and respiratory therapists.<br><br>Duration of mechanical ventilation (hours); rate of successful weaning; hospital mortality.	Protocol-directed weaning significantly reduced median MV duration (35 h vs 44 h, $p = 0.024$) and increased successful weaning rate (RR = 1.31; 95% CI: 1.15–1.50; $p = 0.039$). No significant difference was observed in hospital mortality (22.3% vs 23.6%, $p = 0.779$).

**Cullen et al., 1997**	USA	Prospective cohort study	Medical, surgical ICUs.	Adult patients (N = 4031).	To evaluate the incidence and characteristics of adverse drug events (ADEs) in ICUs compared with non-ICU hospital settings.	Monitoring of Adverse Drug Events (ADEs) and potential ADEs via stimulated self-report by nurses/pharmacists and daily chart review.<br><br>ADE rates per 1,000 patient-days;<br>severity of events;<br>length of stay; hospital charges.	ICU ADE rate was 19 events per 1,000 patient-days, nearly twice that of non-ICUs ($p < 0.01$). Medical ICU had higher ADE rates (25/1,000) compared to surgical ICU (14/1,000, $p < 0.05$). After adjusting for medication load, no difference remained between ICU and ward ADE rates. ICU ADEs were more severe and associated with longer stays and higher costs.

Legend. This table presents the descriptive summary of the 147 studies included in the preliminary scoping review conducted prior to the Delphi consensus process. For each study, key methodological and contextual variables were extracted according to predefined criteria, including: author and year of publication, country, study design, ICU setting, participant profile, study aim, nursing interventions or metrics examined, and main outcomes reported. This structured dataset supported the mapping, classification, and synthesis of nursing-sensitive patient outcomes (NSPOs). Notes. *SD – Standard Deviation*: measure of variability around the mean; *IQR – Interquartile Range*: variability between the 25th and 75th percentile; *Median* – Midpoint of the distribution; *Mean* – Arithmetic average; *Range* – Minimum and maximum values; *p* or *p-value* – Probability value indicating statistical significance; *α (alpha)* – Significance threshold (commonly 0.05); *t-test* – Student's t-test for comparing means; *χ² (Chi-square)* – Test assessing associations between categorical variables; *z-test* – Standardised test statistic for proportions/large samples; *F-test* – Test used in ANOVA to compare variances; *OR – Odds Ratio*: relative odds of an event occurring; *aOR – Adjusted Odds Ratio*: OR adjusted for confounders; *RR – Risk Ratio*: relative risk between groups; *aRR – Adjusted Risk Ratio*; *HR – Hazard Ratio*: comparison of hazard rates over time; *aHR – Adjusted Hazard Ratio*; *CI – Confidence Interval*: range estimating the true value with a given level of confidence (usually 95%); *κ (Kappa) – Cohen's Kappa*: agreement between raters beyond chance; *CV – Coefficient of Variation*: relative variability; *r* – Pearson correlation coefficient (linear correlation).
