# Supplementary material for: Development of a Core Set of Nursing-Sensitive Patient Outcomes in Intensive Care Units: A Delphi Consensus Study
Source: Clin Pract. 2026 Apr 30;16(5):89. doi: 10.3390/clinpract16050089 (PMC13206322; doi:10.3390/clinpract16050089)
Supplement: Supplementary file 1 [file clinpract-16-00089-s001.zip › Table S3. Complete dataset of the Delphi consensus process.pdf]

Table S3. Complete dataset of the Delphi consensus process.

NURSING-SENSITIVE PATIENT OUTCOMES	ROUND 1									ROUND 2									Round 1 vs Round 2					CONSENSUS MEETING	FINAL DECISION
	1. % (n.)	2. % (n.)	3. % (n.)	4. % (n.)	5. % (n.)	Overall % (n.)	Median	IQR	Consensus for inclusion	1. % (n.)	2. % (n.)	3. % (n.)	4. % (n.)	5. % (n.)	Overall % (n.)	Median	IQR	Consensus for inclusion	Grand mean %	Δ Median	Δ IQR	Kruskal-Wallis test	ICC (2,1)		
1. ***Accidental falls***	0 (0)	11 (4)	27 (10)	40 (15)	22 (8)	62 (23)	4	1	No	0 (0)	6 (2)	27 (8)	50 (15)	17 (5)	67 (20)	4	1	No	64	0	0	0.034	0.81	*No consensus*	*Excluded*
2. ***Unplanned extubations***	0 (0)	3 (1)	16 (6)	51 (19)	30 (11)	81 (30)	4	1	Yes	0 (0)	3 (1)	29 (9)	45 (14)	23 (7)	68 (21)	4	1	No	74	0	0	0.067	0.65	*No consensus*	*Excluded*
3. ***Adverse events***	0 (0)	3 (1)	22 (8)	48 (18)	27 (10)	75 (28)	4	1	Yes	0 (0)	3 (1)	16 (5)	68 (21)	13 (4)	81 (25)	4	1	Yes	78	0	0	0.042	0.78	*Consensus for inclusion*	*Included*
4. ***Mortality***	3 (1)	8 (3)	41 (15)	32 (12)	16 (6)	48 (18)	3	1	No	3 (1)	6 (2)	52 (16)	26 (8)	3 (1)	29 (9)	3	1	No	38	0	0	0.015	0.82	*No consensus*	*Excluded*
5. ***Length of ICU stay***	0 (0)	11 (4)	35 (13)	41 (15)	13 (5)	54 (20)	4	1	No	0 (0)	6 (2)	26 (8)	64 (20)	3 (1)	67 (21)	4	1	No	60	0	0	0.089	0.71	*No consensus*	*Excluded*
6. ***Length of hospital stay***	0 (0)	8 (3)	35 (13)	41 (15)	16 (6)	57 (21)	4	1	No	0 (0)	3 (1)	39 (12)	61 (19)	0 (0)	61 (19)	4	1	No	59	0	0	0.053	0.69	*No consensus*	*Excluded*
7. ***Duration of mechanical ventilation***	0 (0)	11 (4)	30 (11)	46 (17)	13 (5)	59 (22)	4	1	No	0 (0)	3 (1)	29 (9)	54 (17)	13 (4)	67 (21)	4	1	No	63	0	0	0.028	0.77	*No consensus*	*Excluded*
8. ***Mismatch of mechanical ventilation***	3 (1)	5 (2)	35 (13)	41 (15)	16 (6)	57 (21)	4	1	No	0 (0)	3 (1)	35 (11)	48 (15)	13 (4)	61 (19)	4	1	No	59	0	0	0.011	0.59	*No consensus*	*Excluded*
9. ***ICU readmission***	0 (0)	16 (6)	30 (11)	38 (14)	16 (6)	54 (20)	4	1	No	0 (0)	3 (1)	32 (10)	13 (4)	23 (7)	38 (11)	4	1	No	46	-1	0	0.060	0.74	*No consensus*	*Excluded*
10. ***Health-related quality of life***	0 (0)	5 (2)	16 (6)	54 (20)	25 (9)	79 (29)	4	0	Yes	0 (0)	3 (1)	23 (7)	48 (15)	26 (8)	74 (23)	4	0	No	76	0	0	0.078	0.81	Stability and reliability	*Included*
11. ***Satisfaction***	0 (0)	5 (2)	19 (7)	57 (21)	19 (7)	76 (28)	4	0	Yes	0 (0)	0 (0)	19 (6)	64 (20)	16 (5)	80 (25)	4	0	Yes	78	0	0	0.032	0.85	*Consensus for inclusion*	*Included*
12. ***Catheter-associated urinary tract infections (CAUTIs)***	0 (0)	3 (1)	13 (5)	57 (21)	27 (10)	84 (31)	4	1	Yes	0 (0)	3 (1)	16 (5)	55 (17)	26 (8)	81 (25)	4	1	Yes	82	0	0	0.046	0.72	*Consensus for inclusion*	*Included*
13. ***Central line-associated bloodstream infections (CLABSI)***	0 (0)	0 (0)	16 (6)	52 (19)	32 (12)	84 (31)	4	1	Yes	0 (0)	0 (0)	16 (5)	48 (15)	36 (11)	83 (26)	4	1	Yes	85	0	0	0.091	0.68	*Consensus for inclusion*	*Included*
14. ***Surgical site infections***	0 (0)	0 (0)	13 (5)	54 (20)	32 (12)	86 (32)	4	1	Yes	0 (0)	3 (1)	19 (6)	58 (18)	19 (6)	77 (24)	4	1	Yes	81	0	0	0.049	0.66	*Consensus for inclusion*	*Included*
15. ***Ventilator-associated pneumonia***	0 (0)	5 (2)	22 (8)	38 (14)	35 (13)	73 (27)	4	2	No	0 (0)	0 (0)	26 (8)	45 (14)	29 (9)	74 (23)	4	2	No	73	0	0	0.058	0.63	*No consensus*	*Included*
16. ***Airway secretion clearance***	0 (0)	3 (1)	24 (9)	49 (17)	24 (9)	73 (26)	4	1	No	0 (0)	0 (0)	16 (5)	42 (13)	42 (13)	84 (26)	4	1	Yes	78	0	0	0.025	0.79	*Consensus for inclusion*	*Included*
17. ***Alterations vital parameters***	0 (0)	8 (3)	43 (16)	35 (13)	13 (5)	48 (18)	4	1	No	0 (0)	6 (2)	58 (18)	23 (7)	10 (3)	33 (10)	3	1	No	40	-1	0	0.017	0.81	*No consensus*	*Excluded*
18. ***Pain***	0 (0)	3 (1)	21 (8)	49 (18)	27 (10)	76 (28)	4	1	Yes	0 (0)	0 (0)	19 (6)	64 (20)	16 (5)	80 (25)	4	1	Yes	78	0	0	0.039	0.83	*Consensus for inclusion*	*Included*
19. ***Comfort***	0 (0)	3 (1)	21 (8)	46 (17)	30 (11)	76 (28)	4	1	Yes	0 (0)	0 (0)	16 (5)	68 (21)	16 (5)	84 (26)	4	1	Yes	80	0	0	0.055	0.76	*Consensus for inclusion*	*Included*
20. ***Post-extubation/tracheostomy dysphagia***	0 (0)	30 (11)	27 (10)	24 (9)	19 (7)	43 (16)	3	2	No	3 (1)	29 (9)	32 (10)	23 (7)	6 (2)	29 (9)	3	0	No	36	0	-2	0.082	0.64	*No consensus*	*Excluded*
21. ***Incontinence-associated dermatitis***	0 (0)	5 (2)	13 (5)	60 (22)	22 (8)	82 (30)	4	0.75	Yes	0 (0)	0 (0)	16 (5)	74 (23)	3 (10)	77 (24)	4	0.75	Yes	79	0	0	0.048	0.69	*Consensus for inclusion*	*Included*
22. ***Pressure injuries***	0 (0)	3 (1)	11 (4)	49 (18)	38 (14)	87 (32)	4	0	Yes	0 (0)	3 (1)	13 (4)	68 (21)	16 (5)	84 (26)	4	-1	Yes	85	0	-1	0.027	0.82	*Consensus for inclusion*	*Included*
23. ***Thromboprophylaxis***	0 (0)	16 (6)	35 (13)	30 (11)	19 (7)	49 (18)	3	1	No	0 (0)	3 (1)	48 (15)	39 (12)	10 (3)	49 (15)	3	1	No	49	0	0	0.073	0.66	*No consensus*	*Excluded*
24. ***Bowel dysfunction***	0 (0)	5 (2)	19 (7)	52 (19)	24 (9)	76 (28)	4	0	Yes	0 (0)	0 (0)	16 (5)	71 (22)	13 (4)	84 (26)	4	0	Yes	80	0	0	0.012	0.80	*Consensus for inclusion*	*Included*
25. ***Altered blood glucose levels***	0 (0)	5 (2)	30 (11)	49 (18)	16 (6)	65 (24)	4	1	No	0 (0)	3 (1)	26 (8)	58 (18)	13 (4)	71 (22)	4	1	No	68	0	0	0.038	0.75	*No consensus*	*Excluded*
26. ***Altered nutritional status***	0 (0)	11 (4)	40 (15)	41 (15)	8 (3)	49 (18)	4	1	No	0 (0)	6 (2)	32 (10)	52 (16)	10 (3)	62 (19)	4	1	No	55	0	0	0.043	0.78	*No consensus*	*Excluded*
27. ***Altered oral mucosal status***	0 (0)	0 (0)	22 (8)	73 (27)	5 (2)	78 (29)	4	0	Yes	0 (0)	0 (0)	23 (7)	71 (22)	6 (2)	77 (24)	4	0	Yes	77	0	0	0.079	0.71	*Consensus for inclusion*	*Included*
28. ***Altered ocular health status***	0 (0)	0 (0)	8 (3)	79 (29)	13 (5)	91 (34)	4	0	Yes	0 (0)	0 (0)	13 (4)	81 (25)	6 (2)	87 (27)	4	-1	Yes	89	0	-1	0.088	0.70	*Consensus for inclusion*	*Included*
29. ***Altered hygiene status***	0 (0)	0 (0)	5 (2)	67 (25)	28 (10)	95 (35)	4	1	Yes	0 (0)	3 (1)	6 (2)	68 (21)	23 (7)	91 (28)	4	1	Yes	93	0	0	0.051	0.72	*Consensus for inclusion*	*Included*

30.	***Altered functional status***	0 (0)	8 (3)	25 (9)	54 (20)	14 (5)	68 (25)	4	1	No	0 (0)	6 (2)	26 (8)	55 (17)	13 (4)	68 (21)	4	1	No	68	0	0	0.036	0.74	*No consensus*	*Excluded*
31.	***Altered sleep quality***	0 (0)	11 (4)	27 (10)	43 (16)	19 (7)	62 (23)	4	1	No	0 (0)	6 (2)	32 (10)	45 (14)	16 (5)	61 (19)	4	1	No	61	0	0	0.028	0.80	*No consensus*	*Excluded*
32.	***Altered cognitive status***	0 (0)	11 (4)	35 (13)	35 (13)	19 (7)	54 (20)	3	1	No	0 (0)	16 (5)	35 (11)	39 (12)	6 (2)	45 (14)	2	1	No	49	-1	0	0.019	0.83	*No consensus*	*Excluded*
33.	*Delirium*	3 (1)	14 (5)	19 (7)	43 (16)	22 (8)	65 (24)	4	1.5	No	0 (0)	13 (4)	32 (10)	29 (9)	26 (8)	54 (18)	4	0.5	No	59	0	-1	0.014	0.85	*No consensus*	*Excluded*
34.	***Anxiety***	0 (0)	11 (4)	19 (7)	51 (19)	19 (7)	70 (26)	4	1.5	No	0 (0)	6 (2)	23 (7)	45 (14)	26 (8)	71 (22)	4	0.5	No	70	0	-1	0.045	0.79	*No consensus*	*Included*
35.	***Family participation in care***	0 (0)	11 (4)	27 (10)	49 (18)	13 (5)	62 (23)	4	1	No	0 (0)	6 (2)	26 (8)	55 (17)	16 (5)	71 (22)	4	1	No	67	0	0	0.057	0.73	*No consensus*	*Excluded*

Legend. The Table riported complete dataset of the Delphi consensus process for all 35 nursing-sensitive patient outcomes (NSPOs). For each outcome, absolute frequencies and percentages for the five-point Likert scale are reported for both Delphi rounds, with values ≥4 representing responses above the predefined consensus cut-off. The table includes overall agreement (scores 4–5), median and interquartile range (IQR) for each round, inter-round stability measures (ΔMedian, ΔIQR), and the final consensus decision. Between-group differences were assessed using the Kruskal–Wallis test, and reliability was evaluated using the two-way random-effects Intraclass Correlation Coefficient (ICC 2,1). This dataset provides a transparent and complete representation of the expert ratings used to determine the final NSPOs core set.
