# Supplementary material for: Development of a Core Set of Nursing-Sensitive Patient Outcomes in Intensive Care Units: A Delphi Consensus Study
Source: Clin Pract. 2026 Apr 30;16(5):89. doi: 10.3390/clinpract16050089 (PMC13206322; doi:10.3390/clinpract16050089)
Supplement: Supplementary file 1 [file clinpract-16-00089-s001.zip › Table S4. Estended set of Nursing Sensitive Patient Outcomes (NSPOs) with conceptual origins and typical metrics..pdf]

Table S4. Estended set of nursing-sensitive patient outcomes (NSPOs) with conceptual origins and typical metrics.

Domain	Brief operational definition	Examples of raw outcome expressions / metrics from primary studies	NSPO
Safety	Unplanned falls occurring during ICU stay, with or without injury, among patients under nursing supervision.	Fall incidence; number of falls per 1,000 patient-days; proportion of patients with ≥1 fall; falls with injury.	Accidental falls
Safety	Removal of an endotracheal tube or tracheostomy cannula not prescribed by the clinical team (self-extubation or accidental).	Rate of unplanned extubations per 100 ventilated days; % of patients with unplanned extubation; need for reintubation; associated complications.	Unplanned extubations
Safety	Unintended or preventable events compromising patient safety, including medication errors, incorrect procedures, or device-related complications..	Global Trigger Tool adapted for ICU (severity algorithm); medication errors; device-related events; number of reported incidents, Naranjo's algorithm.	Adverse events
Safety	Death occurring during ICU or hospital stay, potentially influenced by quality and timeliness of nursing care.	ICU mortality; in-hospital mortality; 28-day or 90-day mortality; risk-adjusted mortality indices.	Mortality
Clinical	Number of days spent in the ICU, reflecting severity, complications and efficiency of care.	ICU length of stay (days); prolonged ICU stay; median ICU stay; ICU-free days at 28 days	Length of ICU stay
Clinical	Total duration of hospitalisation, including and beyond the ICU episode.	Hospital length of stay (days); prolonged hospitalisation; ward readmission delaying discharge.	Length of hospital stay
Clinical	Time spent on invasive mechanical ventilation, from intubation to successful weaning or death.	Ventilator days; prolonged ventilation (>7, >14 days); ventilator-free days at day 28; time to successful extubation.	Duration of mechanical ventilation
Clinical	Inadequate synchrony between patient effort and ventilator support, with potential impact on comfort and outcomes.	Frequency PVA events; double-triggering episodes; duration of mechanical ventilation.	Mismatch of mechanical ventilation
Safety	Unplanned return to the ICU after discharge to a lower level of care within a defined time frame.	ICU readmission within 48–72 h; ICU readmission rate per 100 discharges; early versus late readmission.	ICU readmission
Perceptual	Patient's perception of physical, psychological, and social well-being during ICU stay, as influenced by nursing care.	HRQoL scores (SF-36, SF-36, EQ-5D); physical and mental component summaries.	Health-related quality of life
Perceptual	Patient's overall evaluation of nursing care received, reflecting perceived competence, responsiveness, and empathy.	Satisfaction scores on validated questionnaires; satisfaction with information; satisfaction with nursing support; global rating of care.	Satisfaction
Safety	Presence of urinary tract infection associated with the use of an indwelling urinary catheter, confirmed by clinical and microbiological criteria.	CAUTI incidence per 1,000 catheter-days; proportion of patients with CAUTIs; time to first CAUTI episode.	Catheter-associated urinary tract infections (CAUTIs)

Safety	Laboratory-confirmed bloodstream infection occurring in a patient with a central venous catheter, not related to another infection source.	CLABSI rate per 1,000 catheter-days; number of CLABSI episodes; CLABSI-free survival, infection score (ESCID).	Central line-associated bloodstream infections (CLABSI)
Safety	Development of wound infection following a surgical procedure, characterized by inflammation, exudate, or dehiscence.	SSI/SSTI incidence; superficial versus deep SSI; SSI per surgical patient; wound infection rate.	Surgical site infections
Safety	Pneumonia developing in patients receiving invasive mechanical ventilation, according to clinical and microbiological criteria.	VAP rate per 1,000 ventilator-days; early vs late VAP; proportion of ventilated patients with VAP.	Ventilator-associated pneumonia
Clinical	Airway management in maintaining patency and promoting the clearance of respiratory secretions	Incidence of retained secretions; frequency of suctioning; Observational checklist of procedural steps; episodes of airway obstruction.	Airway secretion clearance
Clinical	Deviations from normal or target ranges in vital signs that may be influenced by monitoring, titration and nursing interventions.	Episodes of hypotension/hypertension; tachycardia/bradycardia; hypoxia (SpO_2); frequency of alarm events.	Alterations vital parameters
Clinical	Patient's reported or observed level of comfort following nursing interventions aimed at controlling pain intensity.	Pain scores (NRS, VAS, BPS, CPOT).	Pain
Perceptual	Patient's perceived state of physical and psychological ease resulting from appropriate nursing interventions.	Comfort scale scores (ICU-ICUESS; GCS), patient-reported comfort; comfort-related items (thermal comfort, positioning, noise, light).	Comfort
Clinical	Swallowing disorders occurring after extubation or in patients with tracheostomy, with implications for aspiration risk and nutrition.	Swallow test after extubation or tracheostomy, Incidence of post-extubation dysphagia and its severity staging	Post-extubation/tracheostomy dysphagia
Safety	Alteration of skin integrity due to prolonged exposure to moisture from urine or feces, leading to erythema or erosion.	Prevalence of IAD; IAD severity scores; new-onset IAD during ICU stay; time to IAD resolution.	Incontinence-associated dermatitis
Safety	Occurrence of localised skin and tissue damage resulting from sustained pressure or impaired mobility.	Pressure injury incidence/prevalence; staging of lesions score (PUKT); Incidence of device-related pressure injuries.	Pressure injuries
Safety	Adequacy of measures aimed at preventing venous thromboembolism in ICU patients.	Documented DVT events; clinical assessment; laboratory monitoring (D-dimer levels).	Thromboprophylaxis
Functional	Regularity, continence, and efficiency of bowel elimination maintained through individualized nursing care.	Constipation incidence; time to first bowel movement; diarrhoea episodes; Stool containment grade.	Bowel dysfunction
Clinical	Deviations from target glycaemic ranges and associated nursing management of insulin therapy and nutritional support.	Blood glucose levels (mg/dL and mmol/L); episodes of hypoglycaemia/hyperglycaemia; time in target range; glycaemic variability.	Altered blood glucose levels

Clinical	Impairment or improvement in nutritional condition during ICU stay, influenced by feeding strategies and nursing surveillance.	BMI; time to initiation of EN (hours); adequacy of caloric/protein intake; weight change; duration of enteral/parenteral nutrition.	Altered nutritional status
Functional	Integrity and moisture of the oral mucosa and associated structures maintained despite risk factors such as xerostomia, mechanical ventilation, or poor hygiene.	Oral assessment scores (OHAT, BOAS, R-THROAT); presence of lesions, ulcerations, thrush; mucosal dryness; oral care frequency.	Altered oral mucosal status
Functional	Compromise of ocular integrity or function related to lagophthalmos or poor production of the typical tear film	Incidence of OSD; dry eye signs; Fluorescein eye stain test, Schirmer test; NANDA-I Taxonomy II criteria; Schirmer test (<10 mm);	Altered ocular health status
Functional	Personal hygiene, cleanliness and skin care appropriate to the patient's condition and level of dependency	Documentation of hygiene care; frequency of complete bed baths.	Altered hygiene status
Functional	Changes in the patient’s capacity to perform basic motor and self-care activities during and after ICU stay.	Functional status scores (FIM, FSS-ICU, Barthel Index, Sindex); ICU-acquired weakness; dependence in ADLs; mobility level at discharge.	Altered functional status
Perceptual	Impaired sleep continuity, depth and restorative value during ICU stay.	Sleep quality scores (RCSQ) ; number of awakenings; patient-reported sleep disturbance.	Altered sleep quality
Clinical	Declines or fluctuations in cognitive functioning during ICU stay, excluding or beyond acute delirium episodes.	Cognitive assessment scores (MRC, MoCA); ICU-related cognitive impairment; screening for long-term cognitive sequelae.	Altered cognitive status
Clinical	Acute disturbance of consciousness and cognition, often fluctuating, related to illness severity and environmental/therapeutic factors.	Delirium scores (CAM-ICU, ICDSC), hypo/hyperactive delirium incidence.	Delirium
Perceptual	Emotional distress, tension or fear experienced by the patient during ICU stay.	Anxiety scores (HADS, HARS, IES-R, STAI); patient-reported anxiety; episodes of agitation linked to anxiety.	Anxiety
Perceptual	Degree to which family members are informed, involved and engaged in the care process and decision-making.	Researcher-developed satisfaction questionnaire; Family participation score (FINC-NA, PPQ, HCAHP); inclusion in care plans.	Family participation in care

Legend. This table presents the complete set of 35 NSPOs generated during the preliminary phase through the structured process of outcome extraction, semantic clustering, deductive domain mapping, and conceptual consolidation. Each NSPO is allocated to one of Doran’s four domains (safety, clinical, functional, perceptual) [16], and accompanied by its conceptual origin within the literature and the typical metrics used to assess it. The table provides a concise and theory-grounded overview of the outcome set used as the evidence base for Delphi Round 1.
